# The Photosynthetic Bacterium *Rhodopseudomonas palustris* Strain PS3 Exerts Plant Growth-Promoting Effects by Stimulating Nitrogen Uptake and Elevating Auxin Levels in Expanding Leaves

**DOI:** 10.3389/fpls.2021.573634

**Published:** 2021-02-04

**Authors:** Shu-Hua Hsu, Meng-Wei Shen, Jen-Chih Chen, Huu-Sheng Lur, Chi-Te Liu

**Affiliations:** ^1^Department of Agronomy, National Taiwan University, Taipei, Taiwan; ^2^Institute of Biotechnology, National Taiwan University, Taipei, Taiwan; ^3^Agricultural Biotechnology Research Center, Academia Sinica, Taipei, Taiwan

**Keywords:** *Brassica* leafy vegetable, cell proliferation, indole-3-acetic acid, nitrogen use efficiency, plant growth-promoting rhizobacteria

## Abstract

*Rhodopseudomonas palustris* strain PS3, a phototrophic bacterium, was originally isolated from a paddy field located in Taipei city, Taiwan, and showed positive effects on the growth of leafy vegetables. The aim of this study was to clarify the mechanism of the beneficial effects exerted by PS3 on plants. An ineffective *R. palustris* strain, YSC3, isolated from a paddy field located in Yilan County, was used as the negative control for comparative analyses. We cultivated non-heading Chinese cabbage (*Brassica rapa* var. *chinensis*) in 1/2 strength Hoagland hydroponic solution, in which nitrate is the main nitrogen source. We evaluated various plant physiological responses to inoculation with different bacterial inoculants. The N use efficiency (NUE) of PS3-inoculated plants was dramatically higher than that of YSC3-inoculated plants. The nitrate uptake efficiency (NUpE) was significantly elevated in plants treated with PS3; however, no excess nitrate accumulation was observed in leaves. We also noticed that the endogenous indole-3-acetic acid (IAA) levels as well as the cell division rate in the leaves of PS3-inoculated plants were significantly higher than those in the leaves of YSC3-inoculated plants. We examined the bacterial transcription of some genes during root colonization, and found that the expression level of IAA synthesis related gene *MAO* was almost the same between these two strains. It suggests that the elevated endogenous IAA in the PS3-inoculated plants was not directly derived from the exogenous IAA produced by this bacterium. Taken together, we deduced that PS3 inoculation could promote plant growth by enhancing nitrate uptake and stimulating the accumulation of endogenous auxin in young expanding leaves to increase the proliferation of leaf cells during leaf development.

## Introduction

Plant growth-promoting rhizobacteria (PGPRs) are soil bacteria that inhabit the rhizosphere and usually have positive effects on plant growth due to their potential to influence plant physiology and nutritional conditions (Kloepper et al., 1980). The use of PGPRs is referred to as an environmentally friendly approach because it reduces the input of chemical fertilizers and pesticides and increases crop yields through several diverse mechanisms. A large number of promising PGPR candidates, such as those in the genera *Bacillus, Pseudomonas, Azotobacter*, and *Azospirillum*, have been isolated and characterized for their beneficial effects (Adesemoye et al., 2009; Bishnoi, 2015; Backer et al., 2018; Aeron et al., 2020).

Several studies have indicated that the purple non-sulfur phototrophic bacteria (PNSB) *Rhodopseudomonas* spp. also have several beneficial effects on plant growth. For instance, two strains of *Rhodopseudomonas* spp., namely, KL9 and BL6, which were isolated from river sediment, displayed plant growth-promoting traits on tomato seedlings under axenic conditions (Koh and Song, 2007). Ge and Zhang (2019) reported that *Rhodopseudomonas palustris* G5 could exert beneficial effects on cucumber seedling growth and salt tolerance via indole-3-acetic acid (IAA) and 5-aminolevulinic acid (ALA) production. In addition, a *R. palustris* strain, GJ-22, was investigated for its biocontrol ability of triggering induced systemic resistance (ISR) against tobacco mosaic virus (TMV) in tobacco plants, and this paper also identified the crucial aggregation stage of GJ-22 strain colonization in the initiation of ISR priming in the plant (Su et al., 2019). In our previous study, two IAA-producing strains of *R. palustris* (PS3 and YSC3) were isolated from paddy fields in Taiwan and PS3 strain exerted plant-growth promoting effects in enhancing shoot fresh weight and the plant nitrogen use efficiency (NUE) of Chinese cabbage in soil pot system (Wong et al., 2014). These strains are closely related to each other and have similar genomic structures and compositions (Lo et al., 2018). Although these two strains have many plant growth-promoting genes in common, we found that only PS3 could markedly promote the yields of several leafy vegetables, such as non-heading Chinese cabbage (*Brassica rapa* var. *chinensis*) and crinkle garden lettuce (*Lactuca sativa* var. *crispa*) (Hsu et al., 2015).

N fertilizers are heavily used in agriculture to increase yield, especially for crops with successive cultivation, such as leafy vegetables (Lian et al., 1996). However, it has been estimated that approximately 50-70% of applied N fertilizers are lost from agricultural lands (Good et al., 2004). The excess chemical fertilizer input into farming systems has led to environmental problems, such as nitrate pollution of ground water and eutrophication of aquatic ecosystems by surface runoff of phosphorus (Carpenter, 2005). The most effective approach to reduce chemical fertilizer input and maintain crop yields is to improve the N use efficiency (NUE) of plants (Cassman et al., 2002). NUE is defined as the total dry weight per unit available N from all sources applied in agriculture practice, which is associated with both N uptake efficiency (NUpE; the capacity of plant to acquire N from the environment) and N utilization or assimilation efficiency (NUtE; also called physiological NUE, the fraction of plant-acquired N converted to grain yield or plant biomass) (Masclaux-Daubresse et al., 2010; Han et al., 2015). Nitrogen metabolism in plants involves several steps, including N uptake, transport, assimilation, and remobilization (Luo et al., 2006; Chardon et al., 2012).

Nitrate (NO_3_^–^-N) and ammonium (NH_4_^+^-N) are the major N sources for plant growth. For most agricultural land crops, such as *Brassica* leafy vegetables, nitrate is the most readily assimilated form of nitrogen (Hirel et al., 2007). To adapt to dramatic fluctuations in nitrate concentrations in the environment, plants have evolved two nitrate uptake systems with different affinities: a high-affinity transport system (HATS) and a low-affinity transport system (LATS) (Krouk et al., 2010; Bouguyon et al., 2012). Nitrate transporters have been classified into two families: NRT1 and NRT2 (Okamoto et al., 2003). It is generally considered that the NRT1 family transporters belong to the LATS, acting when the external nitrate concentration is high (>1 mM). On the other hand, the NRT2 family is characterized as an HATS, which is particularly crucial for plants when environmental nitrate provision is limited (<0.2 mM) (Orsel et al., 2002). These nitrate transporters facilitate the optimization and maintenance of plant growth and nutrient acquisition in response to environmental changes.

The yields of leafy vegetables are mainly determined by the number and size of aerial leaves. Leaf number is dependent on shoot meristem size and leaf initiation rate (Huang et al., 2015). Furthermore, leaf size is determined by the rate and duration of cell division and expansion in the process of development (Andriankaja et al., 2012). Within a plant leaf, the region with the highest cell division rate is localized at the leaf base, and cell division arrest occurs in the middle of the leaf lamina (i.e., the transition area). While cell division ceases, cell expansion starts at the tip of the leaf blade (Rodriguez et al., 2010), and the plant hormone auxin plays an important role in this process (Gonzalez et al., 2012).

Vegetables are considered to be the main source of daily nitrate intake by human beings, contributing approximately 75∼80% of the total intake (Hord et al., 2009). Non-heading Chinese cabbage (*B. rapa* var. *chinensis*) is a popular Asian leafy vegetable with a relatively high nitrate concentration (Luo et al., 2006). Increasing the nitrate content of vegetables to high levels potentially enhances the risk of human illness, including gastric cancer, esophageal cancer and methemoglobinemia (Du et al., 2007). When the N supply exceeds the demand, plants may absorb large amounts of nitrate. Excess N supply is considered to be the major cause of nitrate accumulation (Blom-Zandstra, 1989).

Several studies have indicated that the effectiveness of PGPR is not necessarily coupled with their genetic background or *in vitro* characteristics (Ali et al., 2009; Batstone et al., 2017). For instance, Ali et al. (2009) reported that two isolates, *Staphylococcus sp*. CdR-1 and *Bacillus* sp. NpR-1, have similar IAA-production ability but only *Bacillus* sp. NpR-1 exerted beneficial effects on wheat grain yield. As mentioned, *R. palustris* strain PS3 exerts superior beneficial effects on plant growth and nitrogen use efficiency (NUE), however, its genetically similar strain YSC3 was ineffective. To elucidate the mechanisms underlying plant-microbe interactions, we conducted comparative analyses on plant and bacterial responses following the inoculation with the effective (PS3) and the ineffective (YSC3) strains. According to the results, we deduced that the effective inoculant could trigger a more persistent and wider cell proliferation zone at the leaf base via crosstalk between nitrate and auxin signaling to promote plant growth.

## Materials and Methods

### Plant Materials and Growth Conditions

Seeds of the non-heading Chinese cabbage (*B. rapa* var. *chinensis* “Maruba Santoh”) were purchased from Formosa Farming Materials Co., Ltd. (Taipei City, Taiwan). The seeds were soaked in water for 1 h and then germinated on moist paper at 23°C for 1 day. The germinated seedlings were individually transplanted into a sponge moistened with water. 3 days later, the seedlings were moistened with full-strength Hoagland solution (Hoagland and Arnon, 1950). One-week-old seedlings were transferred to a 35-L plastic container filled with half-strength (1/2) Hoagland nutrient solution (NS) (EC 1.2–1.3 dS m^–1^) and hydroponically grown at 25°C with a day/night photoperiod of 12 h/12 h (artificial light source with 210 μmol m^–2^ s^–1^ intensity) and 50-70% relative humidity in a plant factory (College of Bioresources and Agriculture, National Taiwan University) for 17 days. An air pump (AirMac, Taiwan) was applied to maintain a high concentration of dissolved oxygen and for homogeneous mixing in the hydroponic NS. The pH of the NS was adjusted to 6.0 ± 1.0 with diluted H_3_PO_4_ every 4 days.

### Preparation of Bacterial Inoculants

Two *R. palustris* strains, namely, PS3 and YSC3, were cultivated in PNSB broth at 37°C as described by Lee et al. (2016). YSC3 is an ineffective strain that was used as a negative control in this study. Each bacterial broth was adjusted to obtain a suspension containing 1.5 × 10^8^ colony-forming units (CFU) mL^–1^, and 350 mL of an individual inoculant was applied to the 35-L hydroponic container that was filled with 1/2 Hoagland NS. Therefore, the final concentration of the inoculant in the NS was approximately 1.5 × 10^6^ CFU mL^–1^. Within a 17-day cultivation period, we inoculated the bacterial suspension on the first day [designated as 0 days after transplanting (DAT)] and on the seventh day of cultivation (7 DAT). In the non-inoculated tank (50% NS), 350 mL of PNSB medium was added as a mock treatment.

### Bacterial Colonization Analysis

Bacterial colonization on the plant roots was measured by using the plate counting method with PNSB medium containing tetracycline (20 μg mL^–1^). We confirmed that the two tested *R. palustris* strains were able to survive under this concentration of the broad-spectrum antibiotic, whereas the other microbes in the hydroponic solution were eliminated. For each treatment, the solution samples were collected from five different locations across the hydroponic tank, followed by serial dilution with liquid PNSB broth. To determine the colonization population of each *R. palustris* strain on the roots of Chinese cabbage, root samples were harvested and washed twice with phosphate buffer (1 M, pH 7.0). The colonizing bacteria were removed from the root by sonication in phosphate buffer for 30 min. The CFU count was recorded after 48 h of incubation at 37°C. Six replicates were used per treatment for determining the bacterial population in hydroponic solution, and five replicates were used per treatment for root colonization analysis. No colonies were formed on the tetracycline selection plates from the samples (hydroponic solution or roots) of the non-inoculated treatment (50% NS).

### Determination of the Nitrogen Use Efficiency (NUE) of Plants

The NUE of plants was measured after harvesting at 17 DAT. The total N content of plant tissues was determined by the Kjeldahl method (Eastin, 1978). The NUE, NUpE and NUtE of each sample were calculated according to Xu et al. (2012). The formulas used were as follows:

Total N content per plant (mg of N plant^–1^) = N concentration (mg of N g^–1^) x plant dry mass (g)

NUE (g g^–1^) = Plant dry mass (g)/Total N supply (g)

NUpE (g g^–1^) = Total N content of shoot (g)/Total N supply (g)

NUtE (g g^–1^) = Plant dry mass (g)/Total plant N content of shoot (g)

### Total RNA Extraction and Target Gene Expression Analyses by Quantitative Real-Time PCR (qPCR)

For plant gene expression analyses, plant roots were harvested at 8, 11, and 17 day after inoculation with three replicates per treatment. For each replicate, root was flash- freezing with liquid N_2_ and ground, after which total RNA was extracted using TRIzol reagent (Invitrogen, United States) according to the manufacturer’s instructions. For *in vivo* transcriptional analyses of bacteria, the root-colonized bacteria were detached from the root 24 h after the second inoculation (i.e., 8 DAT) with four replicates per treatment. For each replicate, three plant roots were excised and transferred to a 50-mL tube containing 30 mL of RNA-stabilization buffer [Phosphate-buffered saline: RNAprotect Bacteria Reagent (Qiagen), 1: 2 v/v] and vortex for 10 min at room temperature. After the root tissue was removed from the tube, we collected the bacteria cell pellets through centrifugation (10,000 × *g* for 10 min at 25°C). Subsequently, the cell pellets were flash-frozen with liquid nitrogen and homogenized mechanically with metal beats 30 rpm for 30 s by TissueLyser (Qiagen). Total RNA of bacteria was isolated and prepared for qRT-PCR as described previously (Onate-Sanchez and Vicente-Carbajosa, 2008). For *in vitro* gene expression of bacteria in response to hydroponic solution, PS3 and YSC3 (approximately 1.5 × 10^8^ CFU mL^–1^) were inoculated (10%, v/v) into 30 mL of half strength Hoagland’s solution and incubated at 25^o^C and 220 rpm in the dark. Bacterial cells were collected after 24 h of incubation through centrifugation (10,000 × *g* for 10 min at 25^o^C). The RNA extraction was the same as that described above. Total RNA was then treated with TURBO (Invitrogen, Life technologies, United States) and subsequently revers-transcribed by SuperScript III (Invitrogen, Life technologies, United States) with 12–18-mer oligo (dT) or random hexamer primers. Quantitative PCR for detecting the expression of genes was performed using the SYBR Green Real-Time PCR Master Mix Kit (KAPA Biosystems, United States), and the fluorescence intensity was detected by a LightCycler 480 System (Roche, Germany). The real-time PCR conditions were as follows: denaturation at 95°C for 3 min and 45 cycles of 95°C for 10 s, 60°C for 20 s, and 72°C for 1 s. The melting curve was obtained by heating at 95°C for 5 s and 65°C for 1 min. For gene expression data of plant, we used *EF-1-α* as a reference gene. The primer pairs *EF-1-α*, *BjNRT1.1* and *BjNRT2.1* used for quantitative RT-PCR were originated from the papers of Qi et al. (2010); Goel and Singh (2015). Transcript abundance data were normalized to the transcript abundance of the reference gene (*EF-1-α*). The fold change in the expression of target genes in each treatment was calculated using the following equation: 2^–Δ*Ct*^, ΔCt = (Ct target gene – Ct reference gene) treatment. The values are the mean of three replicates ± SE. For bacteria, we used *rpoD* as a reference gene. The primer pairs *rpoD, flagB, fliM, cheA, cheR, MAO*, and *eps* used for quantitative RT-PCR were originated from the paper of Lo et al. (2018). The fold change in the expression of target genes in each treatment was calculated using the following equation: 2^–ΔΔCt^, ΔΔCt = (Ct _target_ – Ct _rpoD_) _Time 24_ – (Ct _target_ – Ct _rpoD) Time 0_. The bacterial cultures of PS3 and YSC3 were collected before inoculation as the time zero samples. The primers used for qRT-PCR were listed in Supplementary Table 1. Varied concentrations of cDNA templates from Chinese cabbage root tissue (61.87 ng-1.93 ng), PS3 (25 ng-25 pg), and YSC3 (25 ng-25 pg) were used for verifying the efficiency of respective primer pairs, and standard curves were plotted from Ct values.

### Determination of Nitrate-Assimilating Enzyme Activities

Assessment of nitrate reductase (NR) activity was performed according to Luo et al. (2006) with minor modifications. Fresh leaf tissue samples (0.5 g) were ground in a prechilled mortar with 9 mL of ice-cold extraction buffer (pH 7.5; 25 mM potassium (K) phosphate, 10 mM cysteine, and 1 mM EDTA), followed by centrifugation at 4,000 rpm for 15 min at 4°C. Next, 0.4 mL of supernatant was added to a reaction mixture [1.2 mL of 0.1 mM K-phosphate buffer (pH 7.5), 0.1 M KNO_3_, and 0.4 mL of 0.25 M NADH]. In the control group, NADH was replaced with 0.4 mL of K-phosphate buffer. The reaction was performed at 25°C for 30 min. The assay was terminated by the addition of 1 mL of 1% (w/v) sulfanilamide in 3 M HCl. The nitrite amount was determined with 1 mL of 0.02% (w/v) N-naphthyl-(1)-dihydrochloride for 15 min at 25°C. The reaction solution was centrifuged at 12,000 rpm for 5 min at 4°C, and the absorbance of the supernatant was measured at 540 nm.

The method used for determining glutamine synthetase (GS) activity was that of O’Neal and Joy (1973). Frozen leaf tissues (1 g) were homogenized in 8 mL of extraction buffer (pH 8.0; 0.01 M Tris-HCl buffer solution containing 1 mM MgCl_2_, 1 mM EDTA, and 10 mM β-mercaptoethanol). The extraction procedures were carried out at 4°C. The homogenate was centrifuged at 15,000 × *g* for 20 min at 4°C. After centrifugation, 0.2 mL of the supernatant was mixed with 0.8 mL of reaction buffer. The reaction buffer composition was as follows: 0.1 M Tris-HCl buffer (pH 7.4) containing 30 mM MgSO_4_⋅7H_2_O, 50 mM L-glutamate, 10 mM ATP, and 20 mM hydroxylamine hydrochloride. The mixture was incubated for 30 min at 30°C. The reaction was terminated by adding 2 mL of stop solution [5 g of FeCl_3_ and 10 g of trichloroacetic acid (TCA) dissolved in 200 mL of 1.5 N HCl]. The mixture was centrifuged at 1,000 × *g* for 20 min, and the absorbance of the supernatant was measured at 540 nm. The soluble protein content for NR and GS activity calculation was measured as proposed by Bradford (1976). Leaf samples were homogenized in a chilled mortar with ice-cold extraction buffer (0.1 M sodium phosphate buffer, pH 7.0) and centrifuged at 12,000 × *g* for 20 min at 4°C. The supernatant was determined by Bradford reagent to measure the soluble protein content.

### Measurement of Nitrate Levels in Plants

Nitrate concentrations in leaves were measured at 8, 11, and 17 DAT. Fresh tissue samples were extracted with ddH_2_O, and the supernatant was used to determine the nitrate concentration with a LAQUAtwin Compact Nitrate Meter B-743 (Horiba, Ltd., Japan) as described by Chang and Chang (2014). For soluble leaf protein analysis (Supplementary Figure 8), leaf samples were homogenized in a chilled mortar with ice-cold extraction buffer [0.1 M sodium (Na) phosphate buffer (pH 7.0)] and centrifuged at 12,000 × *g* for 20 min at 4°C. The supernatant protein concentration was determined by Bradford reagent to measure the soluble protein content (Bradford, 1976).

### Nitrate Uptake

Nitrate uptake was determined by measuring nitrate depletion in the NS. The nitrate concentration was measured according to the method described by Cataldo et al. (1975). We added 0.4 mL of 5% (w/v) salicylic acid-sulfuric acid to 0.1 mL of NS sample. After vortex thoroughly, the mixture was reacted for 20 min at room temperature. Then, 4.5 mL of 4.2 N NaOH was slowly added to the mixture and reacted for 30 min at room temperature. After the mixture was cooled, the absorbance was measured at 410 nm. A standard curve was made with KNO_3_.

### Determination of Leaf Development

Leaf development was determined according to the method described by (Gonzalez et al., 2010). Plants were harvested and dissected into individual leaves to be photographed, as shown in Supplementary Figure 6. We took the third leaf of *B. rapa* for the leaf development studies at the following stages: early emergence (8 DAT), expanded stage (11 DAT) and fully grown stage (15 DAT). We applied ImageJ software^1^ to measure the area, length and width of the leaves.

The cell number and size in the third individual leaf at the fully grown stage under different treatments were measured by epidermal impression using clear nail polish (Zhang et al., 2018). The abaxial side of the fresh leaf sample was coated with nail polish for 16 h, and then, the leaf tissue was peeled from the nail polish to reveal the epidermal impression. The epidermal impression was mounted on slides for microscopic analysis (BX51, Olympus, Japan) with ImageJ software. The total cell number of an individual leaf was estimated by calculating the average cell number per unit area in the middle region of a full leaf.

### Histochemical Staining of Cell Plates Formed in Leaf Epidermal Cells

Since cell plates are formed at the end of cell division, the cell division rate was estimated by this value during leaf development. Methyl blue, which can be visualized by fluorescence, was used to stain the β-1,3-glucan in the callose of the cell plates (Nishii et al., 2010). The fresh leaves were fixed with an alcohol-based buffer (95% ethanol: acetic acid, 4:1). After fixation, the leaves were sequentially incubated with 100% ethanol, wash buffer [100% ethanol:0.1 M K_2_HPO_4_ (pH 9.0), 1:1] and 0.1 M phosphate buffer before staining. Finally, the leaf tissues were stained with methyl blue buffer (0.02% methyl blue in 0.1 M phosphate buffer) in the dark for 2 days at 4°C before microscopic analysis. Individual stained leaves were cut perpendicularly at three locations along the leaf vein (divided into four zones as follows: 0-25%, 25-50%, 50-75%, and 75-100% of the distance from the base to the apex). Tissues were mounted on slides, and the cell plate numbers in each section were examined by light microscopy (BX51, Olympus, Japan) under a bright or fluorescent field by use of a U-MWU2 filter set with UV excitation (excitation spectrum, 330 to 385 nm; emission wavelength, 420 nm; Olympus, Japan).

### Analysis of Indole-3-Acetic Acid (IAA) in Leaves of *B. rapa* During Leaf Development

We determined the concentrations of IAA in the leaves of *B. rapa* according to the method proposed by Nishii et al. (2010). The third leaves were harvested at 8, 11, and 15 DAT and ground into powder using a prechilled mortar and pestle with liquid nitrogen. Then, 150 mg of the homogenized tissues was extracted with 50 mM Na-phosphate buffer (pH 7) in the dark at 4°C for 20 min. To validate the concentrations of endogenous IAA, stable isotope-labeled [^13^C_6_]-IAA was added as an internal standard during extraction. Extraction was followed by centrifugation at 13,500 × *g* for 15 min, and 1 mL of the supernatant was adjusted to pH 2.7 with HCl (1 N). The extracted sample was then purified by solid-phase extraction (Oasis^TM^ HLB SPE column, Waters, United States). After sample application, the SPE column was washed with 1 mL of 50% methanol and eluted with 1 mL of 80% ethanol. The eluate was evaporated under vacuum at room temperature and stored at −80°C for 1 day before UPLC-MS analysis (ACQUITY UPLC coupled with a Waters Xevo TQ-S triple quadrupole mass spectrometer; Waters, Milford, United States). The evaporated sample was dissolved in 60 mL of ddH_2_O prior to mass analysis under positive electrospray ionization (ESI) mode for endogenous IAA (m/z, 176 > 130) and [^13^C_6_]-IAA (m/z, 182 > 136). Ten microliters of sample was injected into a reversed-phase ACQUITY UPLC HSS T3 column (1.8 μm particle size, 2.1 × 100 mm, Waters) and eluted with 0.1% acetic acid in methanol and 0.1% acetic acid in water with a 20-min gradient program at a flow rate of 0.3 mL min^–1^ at a column temperature of 30°C. Chromatograms were analyzed and processed using MassLynx version 4.1 and TargetLynx software (Waters Corp.). For each sample, the quantification of endogenous IAA consisted of at least three biological replicates, which in turn consisted of four technical replicates each.

### Statistical Analysis

We applied Student’s *t*-test to determine the difference between two group means data. One-Way ANOVA (Parametric) was conducted followed by Tukey Honest Significant Differences (*p* < 0.05) to compare the means data derived from more than two treatment groups per timepoint. Prior to application of ANOVA, the normality and the homogeneity of variances of all data were checked by Shapiro-Wilk normality test and Levene’s Test. All analyses were performed using R 3.5.1 (R Core Team, 2018).

## Results

### Effects of *R. palustris* Inoculants on Plant Growth

**FIGURE 1:**
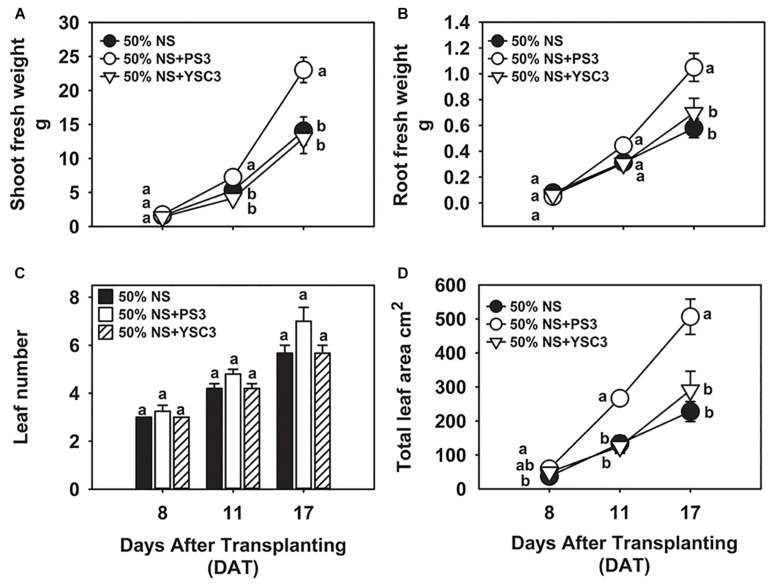
Effects of *R. palustris* inoculants on plant growth. We monitored the growth of *B. rapa* inoculated with two *R. palustris* strains (PS3 and YSC3) in a hydroponic system. The fresh weights of shoots **(A)** and roots **(B)** were measured at 8, 11 and 17 DAT. The number of leaves **(C)** and total leaf area **(D)** were determined at each stage. Data represent means ± standard errors (SEs). Shoot/root fresh weight: *n* = 12; leaf number/area: *n* = 6. Per timepoint, different letters indicate significant differences between treatments (one-way ANOA followed by Tukey’s test; *P* < 0.05).

In our preliminary trials, we noted that the biomass of Chinese cabbage was rapidly increased by the second week of cultivation (later than 8 DAT, i.e., 24 h after the second inoculation at 7 DAT). For the plant growth experiment, we harvested the plant samples at 8, 11, and 17 DAT. As shown in Figure 1A, the biomass of the shoot (fresh weight) under PS3 treatment was significantly higher than that under the other treatments during the cultivation period (from 8 to 17 DAT). At the harvesting time (17 DAT), the shoot biomass of the PS3 treatment group was 82% greater than that of the non-inoculation control group (50% NS) and 77% greater than that of the YSC3 treatment group (Figure 1A). Likewise, the root biomass of the PS3-inoculated plants was also greater than that of the control plants or of the YSC3-inoculated plants during the whole growing period (Figure 1B). Nevertheless, we noticed that the morphology of the root architecture in the PS3-inoculated plants was similar to that in the YSC3-inoculated plants (Supplementary Figure 1). These *R. palustris*-treated plants showed numerous swollen bulbs on the tips of root hairs, which possessed many second- and third-order lateral roots. On the other hand, there were almost no first-order lateral roots in the control plants (Supplementary Figure 1). In addition, we also determined the root length for individual treatments and found that the average primary root length for the PS3 and YSC3 treatments was dramatically shorter than that for the control (Supplementary Figure 2).

In general, an increase in shoot biomass is associated with a higher leaf number or leaf area. As shown in Figure 1C, there was no significant difference in the leaf number among the treatment groups at the respective sampling times. However, we noted that inoculation with PS3 resulted in a 100 and 113% increase in leaf area at 11 DAT and a 123% and 74% increase at 17 DAT in comparison with the leaf areas of the 50% NS and 50% NS + YSC3 treatment groups, respectively (Figure 1D). The bioassay of plant growth was repeated three times, and we found that the effects on leaf and root morphology and biomass of the PS3-/YSC3-inoculated plants were consistent in respective experiments (Supplementary Figures 3, 4). Bacterial colonization on the root and in NS were also monitored during plant cultivation, and the population of PS3 was significantly higher than that of YSC3 on the root and in the solution (Supplementary Figure 5).

### PS3 Enhanced NUE by Stimulating N Uptake Efficiency (NUpE)

**FIGURE 2:**
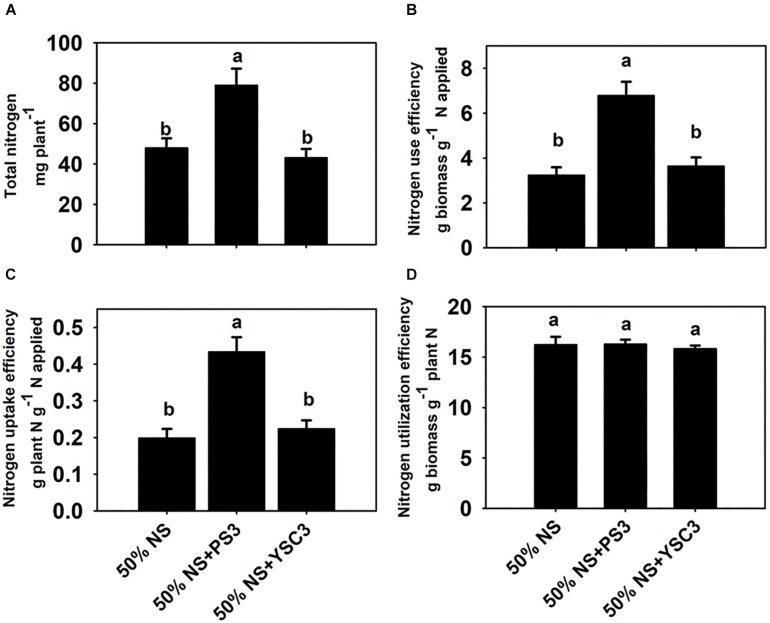
Nitrogen use efficiency of *B. rapa* (17 DAT) under different treatments. **(A)** Total nitrogen content in whole plants. **(B)** Nitrogen use efficiency, NUE. **(C)** Nitrogen uptake efficiency, NUpE. **(D)** Nitrogen utilization efficiency, NUtE. Bars indicate means ± SEs from six biological replicates. Different letters indicate significant differences between treatments (one-way ANOA followed by Tukey’s test; *P* ≤ 0.05).

As shown in Figure 2A, the total N content in the non-inoculation control group (50% NS) at 17 DAT was 47.9 ± 10.6 mg per plant. After inoculation with PS3, the total N content was 78.9 ± 8.2 mg, whereas it remained at almost the same level (43.1 ± 4.4 mg per plant) after YSC3 inoculation. As shown in Figure 2B, the NUE value (17 DAT) of the 50% NS + PS3 group was significantly higher than those of the control group (50% NS) and the 50% NS + YSC3 group. For every gram of N applied under the PS3 treatment, 6.78 ± 0.62 g of dry weight was harvested. In contrast, the harvested dry weight per gram of applied N was only 3.23 ± 0.36 g for the control group (50% NS) and 3.64 ± 0.36 for the 50% NS + YSC3 group. We also found that the NUpE (17 DAT) of the 50% NS + PS3 group was dramatically higher than those of the control group (50% NS) and the 50% NS + YSC3 group (Figure 2C). However, there were no significant difference in NUtE (17 DAT) among the treatment groups (Figure 2D).

### Effects of PS3 Inoculation on Nitrate Uptake and Assimilation in *B. rapa*

**FIGURE 3:**
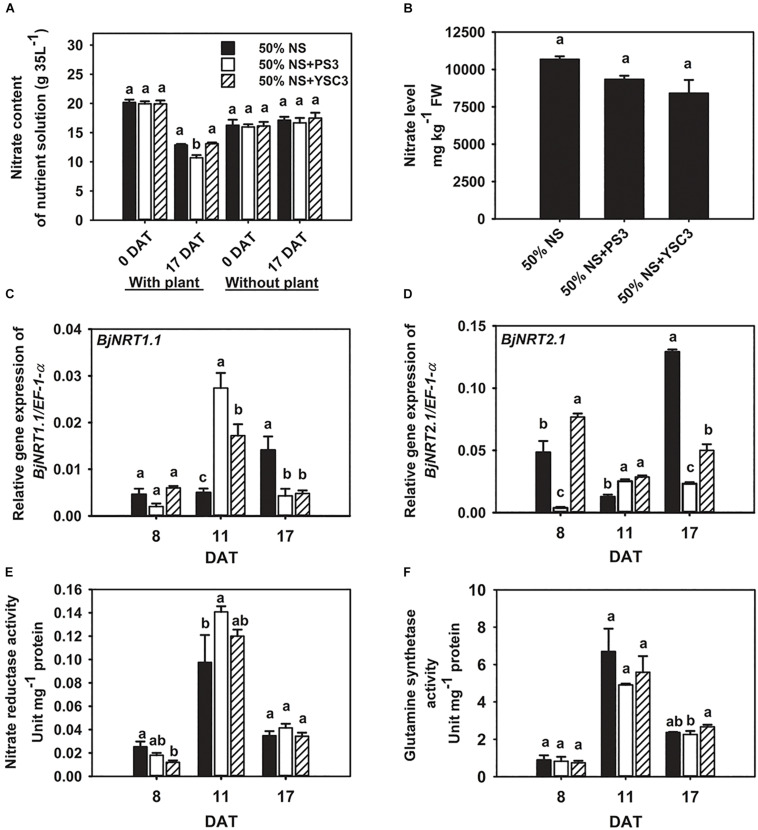
Effects of PS3 inoculation on nitrate uptake and assimilation in *B. rapa*. **(A)** Nitrate content of the NS with or without plants (0 and 17 DAT). **(B)** Nitrate level of plants measured at harvest (17 DAT). The relative expression levels of *BjNRT1.1* **(C)** and *BjNRT2.1* **(D)** were normalized to transcript levels of the housekeeping gene *EF-1-*α by the following formula: 2^–ΔCt^. **(E)** Nitrate reductase activity (8, 11, and 17 DAT). **(F)** Glutamine synthetase activity (8, 11, and 17 DAT). The data represent means ± SEs; Per timepoint, different letters indicate significant differences between treatments (one-way ANOA followed by Tukey’s test; *P* ≤ 0.05; *n* = 4-6).

We cultivated the plants in Hoagland hydroponic solution, where the sole source of N was nitrate-nitrogen. At the beginning of plant cultivation (0 DAT), the same concentration of nitrate was present in the NS for each treatment (∼20 g/35 L, Figure 3A). After 17 days of cultivation (17 DAT), the nitrate content was significantly reduced in the NS of the PS3 treatment group after the Chinese cabbage was harvested (∼10.69 g/35 L, left half in Figure 3A). We also found that the nitrate content in the NS at 17 DAT without plant cultivation was almost the same as that at 0 DAT among the three treatment groups (∼16 g/35 L, right half in Figure 3A). Furthermore, we measured the nitrate level of *B. rapa* at the harvesting stage (17 DAT). We found that PS3 inoculation dramatically increased nitrate uptake in plants; however, it did not lead to excess nitrate accumulation. As shown in Figure 3B, there was no significant difference in the nitrate level of *B. rapa* (17 DAT) among the treatment groups. Furthermore, we also examined the third leaves and found that the nitrate levels in the third leaves over the whole growth period were not increased by inoculation with either PS3 or YSC3 (Supplementary Figure 7).

We further analyzed the transcriptional levels of the genes encoding nitrate transporters to elucidate the bacterial effects on nitrate uptake. The major nitrate transporters of *B. rapa* are designated *BjNRT1.1* and *BjNRT2.1* and are located in the root epidermis (Kiba and Krapp, 2016). Gene expression of *BjNRT1.1* was unchanged after PS3 and YSC3 inoculation at 8 DAT (Figure 3C). The transcript of *BjNRT1.1* was upregulated in the PS3 treatment group at 11 DAT (Figure 3C). On the other hand, the expression of *NRT2.1* was particularly upregulated in the control group at 17 DAT (Figure 3D). In contrast, the expression levels of this gene under PS3 treatment were less than those in the control group during the cultivation period (17 days), except at 11 DAT (Figure 3D). NR activity and GS activity have been proposed as more sensitive indicators of N status in plants than the total N content (de Oliveira Ferreira et al., 2015). To investigate whether the PS3 strain prevents nitrate accumulation by increasing nitrate assimilation activity, we measured the activities of these two enzymes in the leaves during plant growth. In the early growth stage of *B. rapa* (8 DAT), both the NR and GS activities of the control group were low, and these activities peaked in the rapid-growth stage (11 DAT) and then declined in the late growth stage (17 DAT) (Figures 3E,F). We noticed that NR activity was increased by 14% in the PS3-inoculated plants (11 DAT) and only slightly increased in the YSC3-inoculated plants (Figure 3E). However, there was no significant difference in GS activity among the treatment groups during plant growth (Figure 3F). These data showed that the NUpE of the PS3-inoculated plants was significantly higher than that of the control plants; however, excess nitrate did not accumulate in the leaves. Furthermore, as mentioned, the nitrate assimilation rate of PS3 was not increased (Figures 3E,F). Accordingly, this suggests that the increase in NR activity of PS3-inoculated plants was due to the higher nitrate uptake rate at 11 DAT.

### Effects of PS3 Inoculation on Leaf Shape and Size of *B. rapa*

**FIGURE 4:**
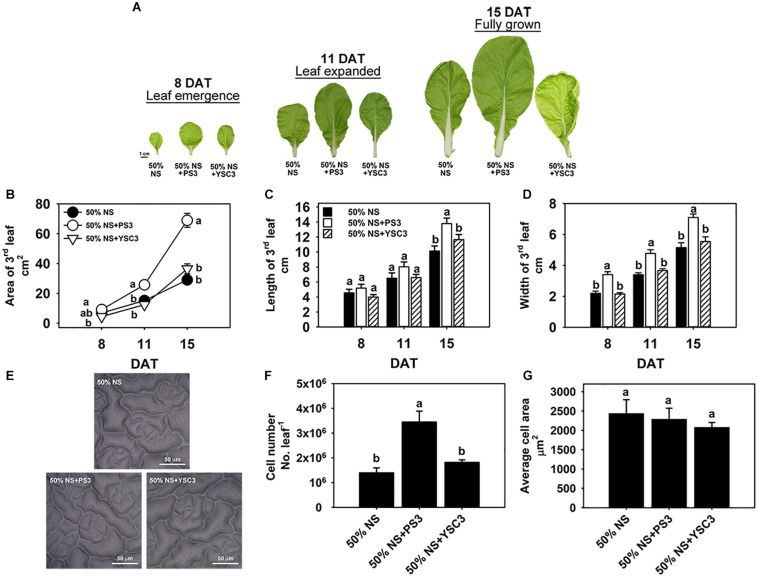
Effects of PS3 inoculation on the leaf shape and size of *B. rapa*. **(A)** Morphology of the third leaf of *B. rapa* harvested at 8, 11, and 15 DAT, which correspond to leaf emergence, expanded and fully grown stages of leaf development, respectively. **(B)** Leaf area. **(C)** Leaf length. **(D)** Leaf width. **(E)** Leaf cell imprint image under a microscope. **(F)** Leaf cell number. **(G)** The average cell size of single cells of a fully opened 3rd leaf was obtained at 15 DAT. The data represent means ± SEs. Leaf area, length and width: *n* = 12; leaf cell number/size: *n* = 6. Per timepoint, different letters indicate significant differences between treatments (one-way ANOA followed by Tukey’s test; *P* ≤ 0.05).

Leaves are the major components of cabbage yield and are responsible for capturing light energy to support plant growth. To further identify the effects of PS3 on leaf morphogenesis, the third leaves were chosen for further examination of leaf development. Figure 4 shows the leaf morphological changes of an individual plant during its development from early emergence (8 DAT) to the fully grown stage (15 DAT). Obviously, the leaves of the PS3-inoculated plants were larger than those of the control or YSC3-inoculated plants (Figure 4A), and we measured the area, length and width of a single leaf. The leaf size gradually increased after the leaf emergence stage from 6.86 ± 0.6 cm^2^ to 29 ± 2.97 cm^2^ at the mature stage in the control treatment group (Figure 4B). Inoculation with PS3 significantly increased the leaf size by 69% and 138% at 11 and 15 DAT, respectively (Figure 4B). We noticed that there was no significant difference in the leaf length among the three treatment groups until 15 DAT (Figure 4C); however, the leaf width in the plants inoculated with PS3 was significantly higher than that in either the control or YSC3-inoculated plants during the entire leaf development period (Figure 4D). These results suggest that the enlargement of the leaf area of *B. rapa* induced by PS3 was mainly due to an expansion in leaf width.

To elucidate the effect of the PS3 inoculant on leaf area enlargement, we further analyzed the cell number and cell size in the leaf surface of a fully expanded leaf (15 DAT) by microscopy (Figure 4E). As shown in Figure 4F, the leaf cell number in the PS3-inoculated plants (3.4 × 10^6^) was superior to those in the control and YSC3-inoculated plants (1.3 × 10^6^ and 1.8 × 10^6^, respectively). However, there was no significant difference in the average cell size among the plants of the three treatment groups (Figure 4G). These data suggest that the promotion of leaf growth by PS3 is mainly an effect on cell division rather than on expansion.

### PS3 Inoculation Increased the Leaf Cell Division Rate

**FIGURE 5:**
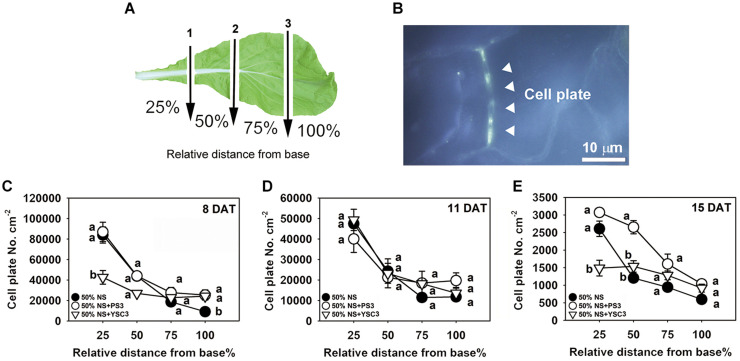
Temporospatial effects of PS3 on leaf cell division of *B. rapa*. **(A)** Schematic diagram of a third leaf that was divided into four zones for cell plate staining. The arrows indicate the cutting sites (1, 2, and 3), which were perpendicular to the central vein. The individual leaves were separated into 25%, 50%, 75%, and 100%, determined according to the relative distance from the leaf base. **(B)** Image under UV excitation of the cell plates stained with methyl blue (1000X, 15 DAT-old PS3-inoculated plants, tip section, 100%). **(C–E)** Cell division rate of the third leaf of *B. rapa* at different developmental stages. The data represent means ± SEs. Per zone, different letters indicate significant differences between treatments (one-way ANOA followed by Tukey’s test; *P* ≤ 0.05) The data are the means of four independent plants and four sample sites for each relative distance.

We further analyzed the cell division rate in the third leaf at each developmental stage (8, 11, and 15 DAT) to elucidate the temporospatial impacts induced by PS3 inoculation. The proliferation of leaf cells follows a longitudinal gradient with basipetal polarity (Donnelly et al., 1999). Accordingly, we divided an individual leaf into four parts according to the relative distance from the base of the leaf and designated the parts as 25, 50, 75, and 100% away from the leaf petiole (Figure 5A). Since cell plates are formed at the end of mitosis during cell division in plants, the density of the cell plates can represent the cell division rate in a whole leaf (Nishii et al., 2010). The cell plates were observed by fluorescence microscopy after staining the leaf with methyl blue and exciting with ultraviolet light. According to the histochemical staining results, the cell division activity declined sharply along the longitudinal axis in a basipetal direction and with the developmental stage (Figures 5A-C). This phenomenon was also reported in the developing leaves of *Arabidopsis thaliana* (Donnelly et al., 1999). The highest cell division rate was achieved in the proximal part of the leaves (i.e., the proliferation zone, 25%), and cell division ceased in the transition zone (i.e., 50 and 75%), while cell expansion occurred in the distal part (i.e., the tip zone, 100%) (Andriankaja et al., 2012). As shown in Figure 5C, the cell plate density in the proliferation zone (25%) of either control or PS3 group leaves (8 DAT) was similar. In contrast, that of the YSC3 group leaves was distinctly reduced in comparison with those of the other groups. At 8 DAT, although the cell plate number was lower in the tip zone than in the other parts (100%), the number in the PS3 or YSC3 group leaves was still significantly higher than that in the control group leaves (1.3- and 1.2-fold, respectively). As shown in Figure 5D, we noticed that the cell plate density at the proliferation zone (25%) in the 11 DAT control group leaves was decreased to approximately half of that in the 8 DAT leaves (Figure 5C). This result indicates that cell division activity was already reduced in the rapid-growth stage (11 DAT). The cell division activity dropped rapidly to ∼25,000 cell plates per cm^–2^ at the transition zone (50% away from the leaf base) and then dropped to ∼12,000 cell plates per cm^–2^ in the tip or expansion zone (100%). In the fully expanded leaves (15 DAT) of control plants, there were fewer than 3,000 cell plates per cm^–2^ in the proliferation zone (25%), followed by ∼1,200 cell plates per cm^–2^ in the transition zone (50%) and fewer than 600 cell plates per cm^–2^ in the expansion zone (100%) (Figure 5E). Strikingly, the cell plates per cm^–2^ value of the 15 DAT PS3 group leaves was almost superior to that of the leaves of the other two treatment groups in all sections, especially in the transition zone, where the cell plates per cm^–2^ value of the PS3 group was 2 times greater than that of the control treatment group (50%) (Figure 5E). However, the value for the 15 DAT YSC3 group leaves was dramatically decreased in the proliferation zone (25%) (Figure 5E). These findings suggest that the cell division rates of the PS3 group leaves could maintain relatively high levels even at the mature stage (15 DAT) (Figure 5E). In contrast, we deduced that the cell division activities of leaves were repressed when the plants were inoculated with YSC3 (Figure 5).

### PS3 Enhanced the Endogenous IAA Level in Leaves

**FIGURE 6:**
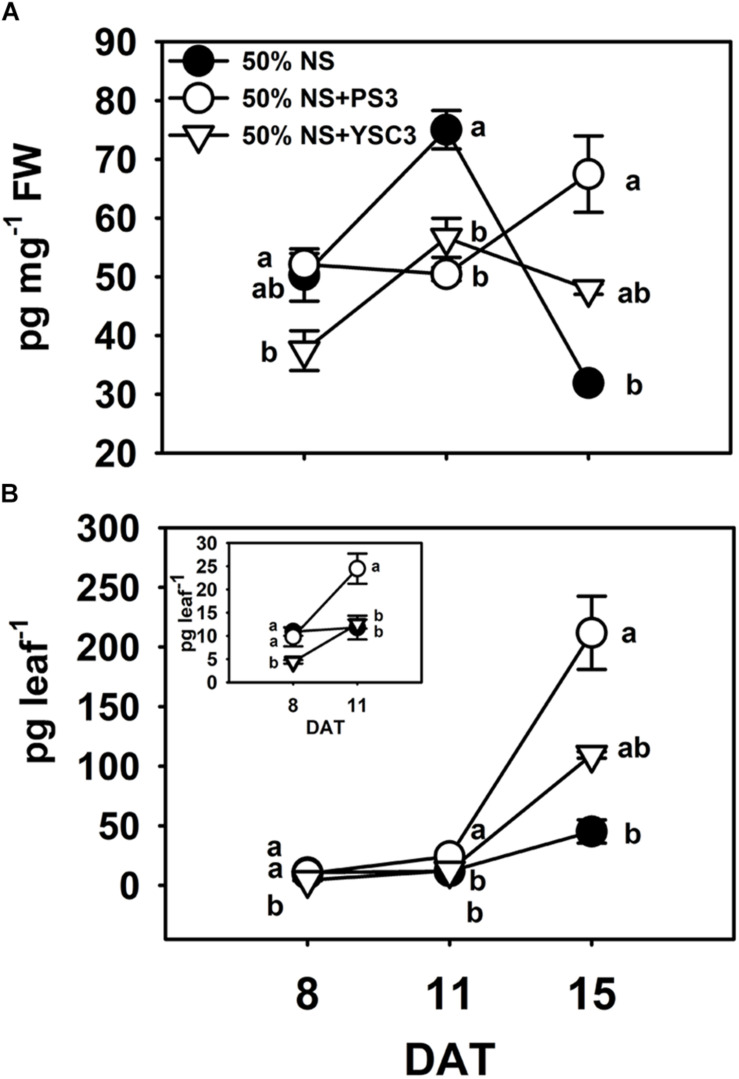
Endogenous indole-3-acetic acid (IAA) levels in leaves. The third leaves of *B. rapa* plants were harvested at 8, 11, and 15 DAT. **(A)** IAA levels were normalized to the fresh weight of leaves. **(B)** The data shown in (A) are represented as IAA level per leaf. The inset highlights the IAA level per leaf at 8 and 11 DAT. Data represent means ± SEs. Per timepoint, different letters indicate significant differences between treatments (one-way ANOA followed by Tukey’s test; *P* ≤ 0.05; *n* = 4).

Indole-3-acetic acid is the major natural form of auxin, which plays important roles in many developmental processes of plant organs (Mroue et al., 2018). We determined the endogenous auxin (IAA) levels in the third leaves. In the control *B. rapa* (50% NS) plants, the maximum concentration of IAA was achieved in the rapid-growth stage (75.01 ± 3.28 pg mg^–1^ FW, 11 DAT), and the concentration decreased to a low level in the fully expanded leaf (31.93 ± 0.51 pg mg^–1^ FW, 15 DAT) (Figure 6A). In contrast, the IAA level in mature PS3-inoculated plants (15 DAT) was elevated to a relatively high level (67.47 ± 6.51 pg mg^–1^ FW) (Figure 6A). Since the distribution of IAA within a leaf is not uniform (Ljung et al., 2001), representing the result as the amount of IAA per fresh weight of leaves may lead to underestimation of any localized increase in individual leaves (Chen et al., 2007). Accordingly, we alternatively calculated the IAA level on a per leaf basis, i.e., were calculated the total IAA level in a respective leaf. As shown in Figure 6B, the endogenous IAA levels in the leaves of the PS3 group plants were higher than those in the leaves of the other two treatment group plants (control and YSC3) at 11 and 15 DAT.

### Transcriptional Modulation of Bacterial Gene Expression Involved in Root Colonization in the Plant-Microbe Interactions

Root colonization of PGPRs is regarded as an essential step to promote plant growth (Marschner, 2012). To elucidate the molecular mechanisms in *R. palustris* PS3 underlying PGPR/plant interactions, we analyzed the bacterial transcription of the genes associated with root colonization in response to inoculation by qPCR. The target genes of *R. palustris* were those related to flagellar motility (*flgB*, *fliM*), chemotaxis (*cheA*, *cheR*), biofilm formation (*eps*) and IAA production (*MAO*), which were derived from the paper of Lo et al. (2018). Since the hydroponic system used for assay was non-gnotobiotic, we confirmed the specificity of all primer sets for bacterial cells collected from the surface of roots in the absence of *R. palustris* inoculation. As shown in Supplementary Figure 9, all the detectable fluorescent signals were derived from the by-products due to primer dimer. In addition, the threshold cycle (Ct) value of the *R. palustris* housekeeping gene *rpoD* was relatively high (32-35) indicating there was no *R. palustris* existed in the background. On the other hand, the Ct values for the amplicon of the target genes in the presence of *R. palustris* were relatively low. For example, those of *flgB* gene for PS3 and YSC3 were approximately 25-26 and 26-27 (Supplementary Figure 9). Accordingly, we proposed that all the primer sets used were specific to determine the target gene expression of *R. palustris* PS3 and YCS3.

**FIGURE 7:**
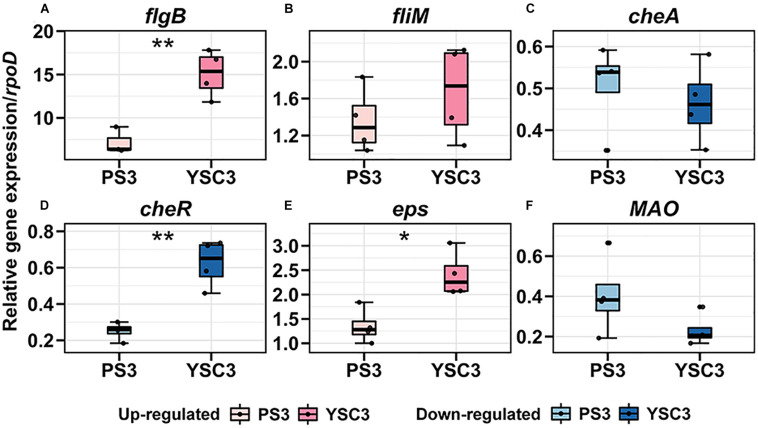
Gene expression pattens of *R. palustris* PS3 and YSC3 during root colonization. Root-colonized bacteria were collected from the root 24 h after the second inoculation (8 DAT). The expression patterns of genes related to **(A,B)** flagella (*flagB, fliM*), **(C,D)** chemotaxis (*cheA, cheR*), **(E)** biofilm formation (*eps*) and **(F)** IAA biosynthesis (*MAO*) were determined by qRT-PCR, respectively. Gene expression was normalized to that of the reference gene *rpoD*. Box plots show the median (horizontal bar within box) and the whiskers extended to the first and third quantiles. Each boxplot represents four biological repeats consisting of bacteria collected from roots of three plants per biological repeat. The asterisk indicates significant differences between PS3- and YSC3-inoculants according to Student’s *t-*test as in *P ≤ 0.05; **P ≤ 0.01.

As mentioned, the cell division rate in the leaf tip at the early leaf emergence stage (8 DAT) was relatively high in the PS3-inoculated plant (Figure 5C). Accordingly, we compared the above individual gene expression of PS3 and YSC3 between pre-and post-inoculation at 8DAT. As shown in Figure 7, the transcripts of the flagellar biosynthesis related genes (*flgB and fliM*) were up-regulated after inoculation in both PS3 (7.2-fold and 1.3-fold) and YCS3 (15-fold and 1.6-fold) during colonization. That of the biofilm related gene (*eps*) was also up-regulated in PS3 and YSC3 with 1.3-fold and 2.4-fold, respectively. On the other hand, those of the chemotaxis related genes (*cheA* and *cheR*), which code for histidine kinase and methyltransferase, were down-regulated in both PS3 (1.9-fold, 3.9-fold) and YSC3 (2.1-fold, 1.6-fold), respectively. That of the IAA-production related gene (*MAO*), which encodes for a monoamine oxidase, was also down-regulated in PS3 and YSC3 with 2.4-fold and 4.3-fold, respectively. Among the genes, the transcripts of *flgB, cheR* and *eps* of YSC3 were significantly higher than those of PS3. In contrast, there were no significant difference in the expression levels of *fliM*, *cheA* and *MAO*.

## Discussion

### Plant Biomass Accumulation Induced by PS3 Was Due to Dramatic Leaf Expansion

*Rhodopseudomonas palustris* PS3 is a soil-borne phototrophic bacterium that has been shown to exert positive effects on plant growth (Wong et al., 2014). In contrast, although strain YSC3 is phylogenetically close to PS3 (Lo et al., 2018), it has no beneficial effects on plant growth. The PS3-treated plants showed obvious increases in shoot and root biomass during growth, especially at the late stage of growth (17 DAT); however, there was no significant difference between the values for YSC3-treated plants and the control plants (50% NS) (Figure 1). It has been widely reported that PGPR inoculants can exert beneficial effects on the growth and yield of edible parts in several leafy vegetables, such as lettuce, cabbage and peppermint (Ruzzi and Aroca, 2015). This effect is attributed to an increase in leaf number and/or leaf size (Bresson et al., 2013; Poupin et al., 2013). As shown in Figure 1C, there was no difference in leaf number among the treatment groups. However, the leaf area was significantly enlarged under treatment with strain PS3 in the rhizosphere (Figure 1D). Accordingly, we deduced that the shoot biomass increases in PS3-treated Chinese cabbage were mainly associated with leaf area expansion rather than elevation in the number of leaf blades.

### NUE of Plants Was Improved by the PS3 Inoculant via Enhancement of Nitrate Uptake

N availability is the major limiting factor for plant growth, and NUE is considered the critical trait for crop production (Xu et al., 2012). As shown in Figure 2B, the value of NUE (17 DAT) of the 50% NS + PS3 group was superior to those of the control group (50% NS) and the 50% NS + YSC3 group (increased 110% and 87%, respectively). This result indicates that the biomass production ability of the PS3 treatment was better than that of the other treatments under the same N supply. NUE is associated with both NUpE and NUtE (Masclaux-Daubresse et al., 2010; Han et al., 2015). The NUpE (17 DAT) of the 50% NS + PS3 group was dramatically higher than those of the control group (50% NS) and the 50% NS + YSC3 group (Figure 2C), whereas there was no significant difference in NUtE (17 DAT) among the treatment groups (Figure 2D). Accordingly, we deduced that the high NUE of the 50% NS + PS3 treatment group (Figure 2A) was mainly due to high NUpE (Figure 2B). Similar phenomena (i.e., enhancement of NUpE) have also been reported in the literature, for example, the application of *Bacillus* spp. in wheat (Adesemoye et al., 2009), application of *Achromobacter* spp. in oilseed rape (Bertrand et al., 2000), application of *Azospirillum brasilense* Sp245 in maize (Dobbelaere et al., 2002), application of *Burkholderia* spp. in grain amaranth (Parra-Cota et al., 2014) and the application of mixed inoculant containing *Azotobacter chroococcum*, *Azospirillum brasilense*, *Pseudomonas fluorescens*, and *Bacillus subtilis* on lettuce in a hydroponic culture system (Aini et al., 2019). These findings indicate that PGPR can induce physiological alterations in plants by affecting the uptake of nutrients.

We determined the residual nitrate content of the hydroponic solution for each treatment after harvest (17 DAT) and found that the residual nitrate content of the PS3 treatment group (10.69 ± 0.46 g 35 L^–1^) was significantly lower than those of the other two treatment groups in the presence of plant cultivation (12.88 ± 0.49 and 13.10 ± 0.59 g 35 L^–1^, respectively, Figure 3A). According to the data derived from the experiments in the absence of plant cultivation (Figure 3A), neither PS3 nor YSC3 altered the nitrate level in the hydroponic solution. Accordingly, we proposed that nitrate in the hydroponic solution was not consumed by the inoculated bacterial strains. Therefore, the plants inoculated with PS3 took up more nitrate from the hydroponic solution than the others, and these data are also consistent with the high NUpE of the 50% NS + PS3 plants shown in Figure 2C.

We further evaluated the activities of the nitrate transporters by analyzing the transcripts of related genes, i.e., *BjNRT1.1* and *BjNRT2.1*, in Chinese cabbage. NRT1.1 is a dual-affinity transporter that plays a dual role in nitrate transport and signaling nitrate (Ho et al., 2009). NRT2.1 is a high-affinity nitrate transporter that plays an important role in N uptake in response to N starvation (Wang et al., 2012). The hydroponic solution used in this study was 1/2 Hoagland NS containing 7.5 mM nitrate, which is defined as a high-N-supply condition. As shown in Figure 3C, the root *NRT1.1* gene was induced dramatically by PS3 inoculation at 11 DAT, which is consistent with the higher NUpE observed in PS3-inoculated plants. The high-affinity nitrate transport system in roots is both substrate inducible and feedback regulated by systemic signals based on the whole-plant N status (Gansel et al., 2001). Mantelin et al. (2006) reported that when *Arabidopsis* was inoculated with a beneficial bacterium, *Phyllobacterium* strain STM196, the transcription of *AtNRT2.1* was downregulated for feedback regulation by increased N content in the shoot. Our data showed that the gene expression of *NRT2.1* was dramatically induced in the control group at the late culture stage (17 DAT, Figure 3D). In contrast, in the PS3-inoculated plants, the expression level of *NRT2.1* was not changed throughout the growing period. Therefore, we propose that the PS3-inoculated plants had sufficient amounts of N for growth and development during the cultivation time.

Chinese cabbage is classified as a plant with high nitrate accumulation (Staugaitis and Dris, 2002). We noticed that although the PS3-inoculated plants took up more nitrate from the NS than those of the other treatments (Figure 3A), they did not accumulate more nitrate in the plant tissue than the plants from the other two treatment group (Figure 3B). It is well known that the nitrate content in plant tissue is negatively correlated with NR and GS activities (Good et al., 2004). However, we found that neither the NR nor GS activity in the PS3-treated plants was significantly higher than those in the plants of the other treatment groups during growth, although the NR activity in the leaves of the PS3 group 11 DAT plants was higher than that in the control group plants (Figure 3C). Accordingly, we deduced that PS3 does not cause a reduction in nitrate accumulation in plants by altering the efficiency of the N assimilation pathway. A recent study reported that when the PGPR *Pseudomonas nitroreducens* strain IHB was used to inoculate *A. thaliana* or *L. sativa* (lettuce), both plants exhibited markedly improved growth, and nitrate uptake was stimulated in the plants; moreover, the shoot nitrate level in the inoculated plants was less than that in the plants without inoculation (Trinh et al., 2018b). The researchers indicated that the plant biomass enhancement observed upon IHB inoculation could offset the high levels of nitrate uptake. Therefore, we speculate that the PS3 inoculant could assist host plants in maintaining relatively low nitrate concentrations by enhancing their growth.

### The PS3 Inoculant Stimulated Endogenous IAA Accumulation and Resulted in Elevation of Cell Cycle Activity in Young Expanding Leaves

As mentioned above, the increases in the shoot biomass of PS3-inoculated Chinese cabbage were mainly due to the enlargement of leaf area (Figure 4). Since leaf growth is associated with the rate and duration of cell proliferation (division) and the extent of postmitotic cell expansion (Andriankaja et al., 2012), we examined the total cell number and average cell size in fully opened third leaves (15 DAT). As shown in Figure 4F, the cell number of PS3-inoculated plants was superior to those of control and YSC3-inoculated plants. However, there was no significant difference in the average cell size among the plants. In contrast, a study reported that inoculation of *Arabidopsis* with a PGPR strain, *Burkholderia phytofirmans* PsJN, resulted in enlargement of leaf area, which was due to cell expansion (Poupin et al., 2013). Taken together, the results suggest that the promotion of leaf growth by PS3 is mainly dependent on cell division rather than on cell expansion. The factors secreted from PS3 as well as their modes of action in the mediation of host cell division remain to be elucidated.

It is known that the timing for the transition from cell proliferation to cell expansion is also a critical determinant of leaf size (Gonzalez et al., 2012). In addition, the cell division activity of leaves gradually declined along the longitudinal axis in a basipetal direction, and the arrest front indicates the switch from proliferation to expansion (Andriankaja et al., 2012). To illustrate the temporal and spatial changes in cell proliferation activity in *B. rapa* leaves, we applied the method of cell plate staining-based imaging in this study. As shown in Figure 5C, we noted that when the plants were inoculated with PS3, the cell division rate (i.e., # of cell plates) in the leaf tip at the early leaf emergence stage (8 DAT) was relatively high. Furthermore, the cell division rates in all four zones (25-100%) of the 15 DAT PS3 group leaves were still higher than those in the leaves of the other two treatment groups (Figure 5E). Strikingly, the value for the PS3 treatment group was almost two times higher than that for the control or YSC3 treatment group at the transition zone (50%), indicating that the cell proliferation arrest front migrated toward the tip direction along the PS3 group leaves. Taken together, the results suggest that PS3 inoculation could trigger a more persistent (longer duration) and wider cell proliferation zone at the leaf base than YSC3 or no inoculation. To date, the phenomena associated with PGPR-induced cell proliferation have mostly been discussed in the context of root tissues. For example, Zamioudis et al. (2013) reported that when *Pseudomonas* sp. strain WCS417 was used to inoculate the roots of *Arabidopsis*, plant growth was promoted, and the cell division rate in the root meristem zone was enhanced via elevation of auxin levels. Until recently, Trinh’s group applied CycB1pro:GUS transgenic lines of *Arabidopsis* to show that PGPR strains were able to induce cell division in leaves (*Paenibacillus pabuli* P7S and *P. nitroreducens*) (Trinh et al., 2018a,b). Nevertheless, our study is the first report of this phenomenon, providing detailed information on the temporal-spatial effects of PGPR on the extension of cell proliferation in shoots, and the molecular mechanisms controlling this phenomenon warrant further study.

Auxin has been demonstrated to play a crucial role in regulating cell proliferation (Perrot-Rechenmann, 2010). We found that the endogenous IAA level in the leaves of the PS3-inoculated plants was significantly higher than in those in the control and YSC3 treatment group plants (Figure 6B). Accordingly, we proposed that the high cell proliferation activity was stimulated by the elevation of endogenous IAA levels in the young expanding leaves of PS3-inoculated plants. Some literature has indicated that there is a positive correlation between endogenous auxin levels in host plants and exogenous auxin production by beneficial microbes (Ali et al., 2009; Mehmood et al., 2019). Nevertheless, the PS3 and YSC3 strains synthesize IAA at almost the same level in the presence of tryptophan (Lo et al., 2018), indicating that exogenous IAA production did not vary between the two *R. palustris* strains near the roots. Furthermore, we also verified the gene expression of the IAA-synthesis related gene *MAO* was at the same level between the two bacteria (i.e., PS3 and YSC3) during their colonization (Figure 7F). Auxin/IAA is known to be associated with root architectural changes via the control of primary root elongation and lateral root formation (Reed et al., 1998; Bhalerao et al., 2002), and the exogenous IAA produced by PGPR has been reported to inhibit primary root elongation and promote the formation of lateral roots (Vacheron et al., 2013). As shown in Supplementary Figure 1, the morphology of the root architecture in the PS3-treated plants was similar to that in the YSC3-treated plants, unlike that in the control plants. This suggests that the lateral root development of Chinese cabbage was influenced by the IAA produced by the *R. palustris* inoculant. In addition, the average primary root length of PS3- or YSC3-inoculated plants was dramatically shorter than that of the control plants (Supplementary Figure 2), indicating that root growth was inhibited by the exogenous IAA produced by the *R. palustris* inoculants, and they had comparable effects.

Auxin molecules can move over short and long distances in plants (Petrasek and Friml, 2009). The auxin flow directions are both basipetal (from the root tip toward the base) and acropetal (from the base toward the root apex) in the roots and are basipetal or lateral in the shoots (Muday and DeLong, 2001; Lewis and Muday, 2009). In plants, the polar auxin transport is mainly mediated by the family of PIN-formed proteins (PINs) (Petrasek and Friml, 2009). It has also been documented that PGPR can affect auxin metabolism and homeosis in host plants (Hayat et al., 2010; Tsukanova et al., 2017). Some beneficial bacteria have been shown to be able to induce the activity or gene expression of the PIN2 transporter, suggesting that PGPRs are able to enhance the upward flow of auxin to the root elongation zone (Felten et al., 2009; Poupin et al., 2016). However, no transport path of auxin from the root to the shoot has been identified, and the source of shoot-accumulated auxin is unlikely to be the root (Muday and DeLong, 2001). Taken together, the results indicate that auxin accumulation in the shoot was systemically induced by root-colonizing PS3, not due to direct transport of auxin from the roots. The factors secreted by PS3 and their mechanisms of action in the mediation of host auxin pathways remain to be elucidated.

### Crosstalk Between Nitrate Uptake and Auxin in Plants Under PS3 Inoculation

As mentioned above, we deduced that PS3 inoculation can promote plant growth by enhancing nitrate uptake and stimulating the accumulation of endogenous auxin. Nitrate is not only a major form of nitrogen but also an essential signaling molecule in plants (O’Brien et al., 2016; Fredes et al., 2019). The effect of nitrate signaling on plant growth and development is associated with auxin (Krouk, 2016). Many studies have indicated that there is multilevel crosstalk between nitrate levels and auxin homeosis in plants (Guan, 2017); hence, we wondered whether nitrate-auxin interactions played a role in our study. We noticed that the nitrate uptake in the roots of PS3-inoculated plants was markedly elevated at 11 DAT (Figure 3C), while the cell division rate in the shoots was dramatically altered in the PS3-inoculated plants at 8 DAT (Figure 5C). According to the timeline, cell proliferation occurred in shoots prior to nitrogen nutrient absorption in roots. Auxin has been demonstrated to play a crucial role in regulating cell proliferation (Perrot-Rechenmann, 2010). As mentioned above, the high cell proliferation activity was supported by the elevation of endogenous IAA levels in the shoots of the PS3-inoculated plants, which was considered to be systemically mediated by root-colonizing PS3. In addition, it has also been demonstrated that shoot-derived auxin is essential for the positive effect of nitrate on lateral root growth, as determined by blocking shoot-to-root auxin basipetal transport with TIBA (2,3,5-triiodobenzoic acid, auxin polar transport protein inhibitor) (Guo et al., 2005). Some studies showed the shoot-derived auxin is import for the beneficial effects of PGPRs. For example, Contesto et al. (2010) reported that *Phyllobacterium brassicacearum* strain STM196 could stimulate the expression of IAA biosynthesis genes in shoots of *Arabidopsis*. Poupin et al.’s (2016) reported that *B. phytofirmans* strain PsJN caused *Arabidopsis* to transport auxin basipetally from shoots to roots. Therefore, we speculated that the shoot-derived auxin in the PS3-inoculated plants was subsequently transported to roots to trigger lateral root development and then stimulate the expression of *BjNRT1.1* for nitrate uptake.

### Differential Expression Events Between PS3 and YSC3 During Root Colonization

Root exudates attract PGPR in the rhizosphere for root colonization, and also provide nutrition for their growth (Badri and Vivanco, 2009). It has been known that several genes related to motility, bacterial chemotaxis and biofilm formation of PGPR were induced by the root exudates (Swamy et al., 2016). In our previous study, we used hydroponic solution supplemented with *B. rapa* root exudate to cultivate the *R. palustris* strains and found that it affected the biofilm formation as well as the relative expression levels of some flagella-related genes (*fliM*, *fliB*, and *fliE*) and chemotaxis-related genes (*cheR, cheW*, and *cheA*) of PS3 and YSC3 (Lo et al., 2018). We noticed that although PS3 produced more biofilm than YSC3 did, there was no difference between them in the expression of biofilm formation-related genes (*fliE* or *exoR* or *eps*). On the other hand, the expression levels of the chemotaxis-related genes (*cheW, cheA*, and *cheR*) of PS3 were significantly higher than those of YSC3 (Lo et al., 2018). In this study, we directly analyzed the transcript levels of these genes while the two *R. palustris* strains were colonized on the root of *B. rapa*. As shown in Figure 7, the transcriptional patterns of these genes were altered in both strains after root colonization. The transcript levels of flagellar motility (*flgB*), chemotaxis (*cheR*), and biofilm formation (*eps*) related genes of YSC3 were significantly higher than those of PS3, and there was no significant difference in the levels of *fliM* and *cheA* (Figure 7). In comparison with the transcripts of bacteria grown in hydroponic solution without *B. rapa* (Supplementary Figure 10), we assumed that the up-regulation of *flgB* gene in the root-colonized YSC3 was due to the stimulation of hydroponic solution, and the differential expressions of *cheR* and *eps* between PS3 and YSC3 were triggered by root exudate. It suggests that PS3 and YSC3 respond differently to nutrient status and the rhizosphere environment of host plant.

The bacterial chemotaxis signal transduction system is a sensory perception system that allows bacteria to move toward favored environments (chemoattractants) or away from unfavored sites (chemorepellents) (Wadhams and Armitage, 2004). The chemotaxis-mediated response to specific compounds in root exudates is essential for root colonization and beneficial functions of PGPR. Feng et al. (2018) have identified several particular chemoeffectors present in root exudates, which trigger chemotaxis mobility and colonization in a well-studied PGPR strain *Bacillus amyloliquefaciens* SQR9. In this study, we found that the transcript levels of the chemotaxis related genes (*cheA* and *cheR*) were down-regulated in both PS3 and YSC3 during root colonization, in which the *cheR* of PS3 was dramatically lower than that of YSC3 (Figures 7C,D). CheR (encoded by *cheR*) is a methyltransferase which is responsible for methylation of methyl-accepting proteins (MCPs) to mediate sensory adaptation and gradient sensing (Spiro et al., 1997) with methylesterase CheB. Its activity is affected by the presence of chemoattractants and chemorepellents. As chemoattractants leave or chemorepellents bind, its activity increases to methylate MCPs, and then activates the histidine kinase CheA (encoded by *cheA*) which leads to tumbling movement by the clockwise (CW) rotation of flagella (Colin and Sourjik, 2017). As mentioned, the transcripts levels of *cheR* in the two tested *R. palustris* strains were down-regulated during root colonization, and that of PS3 was dramatically lower than that of YSC3 (Figure 7D). Accordingly, we assume that PS3 was more sensitive than YSC3 toward chemoattractants or away from chemorepellents that were derived from *B. rapa* root exudates during root colonization. Intriguingly, the relative expression levels of several chemotaxis-associated genes, including *cheA* and *cheR*, were significantly higher for PS3 than for YSC3 upon treatment with root exudates in hydroponic solution (Lo et al., 2018). These *in vitro* data were opposite to the effect *in vivo*. Lopez-Farfan et al. (2019) reported that the transcriptional levels of some chemoreceptor genes in *Pseudomonas putida* KT2440 were induced at low concentration of maize root exudate, whereas those were repressed upon high concentration of root exudate. It remains to be elucidated that whether the chemotactic behavior of the two tested *R. palustris* strains as that of *P. putida* KT2440 response to different concentrations of root exudate on chemoreceptor gene transcript levels. Further experiments are needed, such as identification of the specific components in *B. rapa* root exudate for triggering chemotaxis response, or quantification of the concentrations of the chemoeffectors in the root exudate. Based on the differential transcriptional profiles mediated by PS3 and YSC3 in the rhizosphere, these two strains with close phylogenetic relationship show distinct metabolic regulation and rhizosphere lifestyle which in turn leading different effectiveness on the plant- growth of host plants.

## Conclusion

We deduced that the differences in the concentrations of endogenous IAA between PS3- and YSC3-inoculated plants were due to the differential responses elicited by the compatible and incompatible bacterium-plant interactions. The molecular and cellular mechanisms remain to be elucidated. In summary, when non-heading Chinese cabbage was inoculated with the elite PGPR strain *R. palustris* PS3, its NUE was significantly elevated via enhancement of N uptake. The positive effect on the yield of the edible part was mainly associated with leaf area expansion, which was due to a high cell division rate in the leaf tip at the early leaf emergence stage and due to expansion of the arrest front of the cell division region in leaves at the mature stage. We deduced that the PS3 inoculant could stimulate the accumulation of endogenous auxin in young expanding leaves to trigger a more persistent (longer duration) and wider cell proliferation zone at the leaf base than YSC3 or no inoculant.

## Data Availability Statement

The original contributions presented in the study are included in the article/Supplementary Material, further inquiries can be directed to the corresponding author/s.

## Author Contributions

S-HH, H-SL, and C-TL conceived and designed the study. S-HH and M-WS carried out the experiments and analyzed the data. J-CC assisted with cell division activity analysis and participated in data discussion. S-HH and C-TL wrote the manuscript with input from all authors.

## Conflict of Interest

The authors declare that the research was conducted in the absence of any commercial or financial relationships that could be construed as a potential conflict of interest.

## Footnotes

1 http://rsb.info.nih.gov/ij/
