# Supplementary material for: The Photosynthetic Bacterium Rhodopseudomonas palustris Strain PS3 Exerts Plant Growth-Promoting Effects by Stimulating Nitrogen Uptake and Elevating Auxin Levels in Expanding Leaves
Source: Front Plant Sci. 2021 Feb 4;12:573634. doi: 10.3389/fpls.2021.573634 (PMC7889516; doi:10.3389/fpls.2021.573634)
Supplement: Supplementary file 1 [file Data_Sheet_1.pdf]

Supplementary Table and Figures

Supplementary Table S1 Primer sets used in this study

Genes	Forward primer (5'→3') / Reverse primer (5'→3')	Amplicon size (bp)	Primer efficiency (E)	Slope	Coefficient (R^2)	Reference
EF-1-a	ATACCAGGCTTGAGCATACCG	117	1.93	-3.5	0.98	Qi *et al*., 2010
	GCCAAAGAGGCCATCAGACAA					
BjNRT1.1	CTTCCTCGGAACTTCGTTCATG	100	1.9	-3.6	0.99	Goel and Singh , 2015
	TTGGATAGCGGCGAATATAGC					
BjNRT2.1	AAGAACATGCTTCCCCACTGA	100	2.01	-3.3	0.99	Goel and Singh , 2015
	TCACAACCGAA CAAGGGCTAA					
rpoD	GGTTGGCCTCGACCATTT	77	(2.1, 1.9)*	(-3.2, -3.6)	(0.99, 1)	Lo *et al.* , 2018
	CCGCAAGATCGTCCACTC					
flagB	ATAACAAGGGCGGCTTCC	95	(2.1, 1.99)	(-3.2, -3.35)	(0.99, 0.99)	
	GGCGTAGTCCATCTGGTTG					
fliM	TGCACATCGACATGGAAGA	67	(1.80, 1.81)	(-3.87, -3.89)	(1, 0.99)	
	GGTTCGATCGTGGCGTAT					
cheR	TTGCCGAACTGATCGAAGA	61	(1.9, 2.06)	(-3.6, -3.18)	(0.99, 0.99)	
	AGCATCAGACGTTTGGCATT					
cheA	TCTCGTGACCGTGAAGGAC	122	(1.95, 1.95)	(-3.45, -3.45)	(0.99, 0.99)	
	GGAACTGATGGATTTCCTGGT					
MAO	TGCACGATCTCGAATCCTATC	121	(2.0, 1.99)	(-3.31, -3.35)	(0.99, 0.98)	
	AGCTCGACCACGCAGTTC					
EPS	CGGTCGAAGTGGAATCGTAT	97	(2.0, 1.97)	(-3.36, -3.4)	(0.99, 0.98)	
	GCGCTCTCGACGCTCATA					

*(PS3, YSC3)**

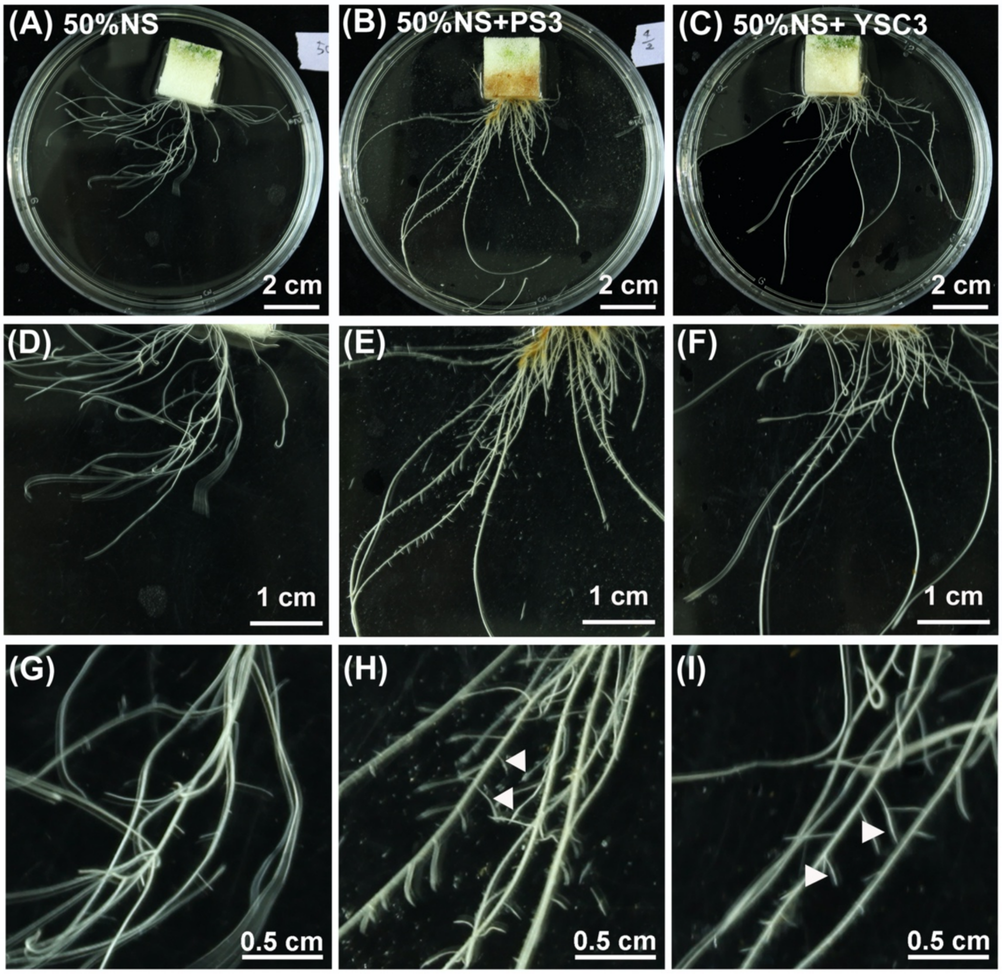

Fig. S1 HSU

Figure S1. Root morphology of *Brassica rapa* after inoculation with *R. palustris* strains. Eight-day-old plants were grown in half-strength Hoagland's nutrient solution (50% NS) with or without *R. palustris* inoculation. The white scale bar represents 2 cm in images A-C. **(A)** Noninoculated control, **(B)** 50%NS+PS3, **(C)** 50%NS+YSC3. Magnified images of the roots: bars in D-F, 1 cm. **(D)** Noninoculated control, **(E)** 50%NS+PS3, **(F)** 50%NS+YSC3. Magnified images of the swollen bulbs on the tips of root hairs (arrows). Bars in G-I, 0.5 cm. **(G)** Noninoculated control, **(H)** 50%NS+PS3, **(I)** 50%NS+YSC3.

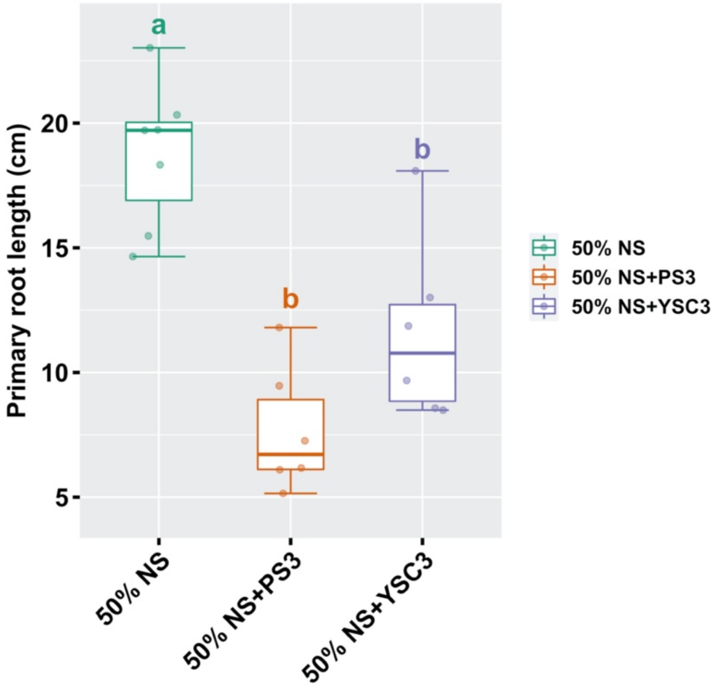

Figure S2. **The effect of *R. palustris* PS3 and YSC3 on the primary root length of *B. rapa.*** The primary root length of the plants was measured at 11 DAT. Box plots show the median (horizontal bar within box) and the whiskers extended to the first and third quantiles with individual sample points (n=6); different letters indicate significant differences between treatments (one-way ANOA followed by Tukey's test; $P \leq 0.05$)

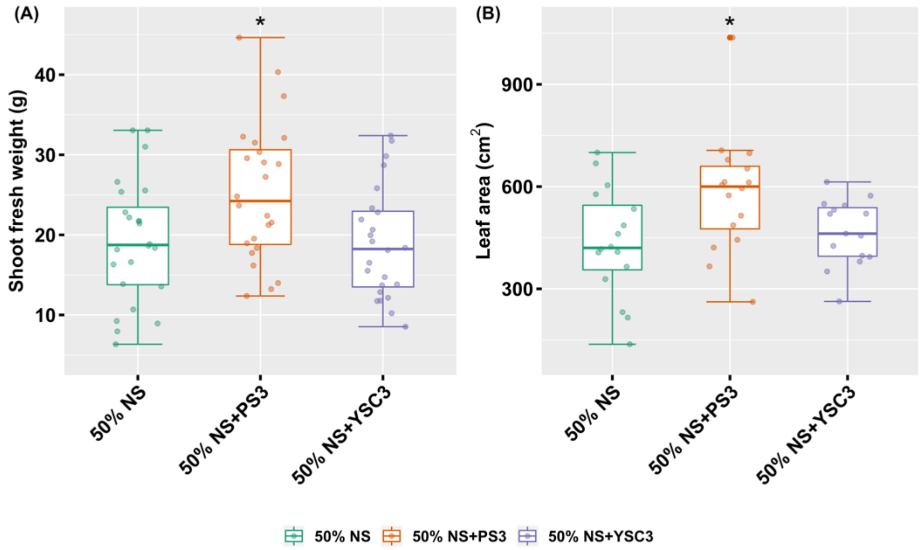

Figure S3. Beneficial effect of the PS3 strain on plant growth (second bioassay). The fresh weights of shoots **(A)** and total leaf area **(B)** were measured at 17 days after treatment. Box plots show the median (horizontal bar within box) and the whiskers extended to the first and third quantiles with individual sample points. Shoot fresh weight: n=24; leaf area: n= 15-16. The asterisk indicates the significant differences between mock- and bacteria-treated plants according to Student's t test as in '*': $P \leq 0.05$.

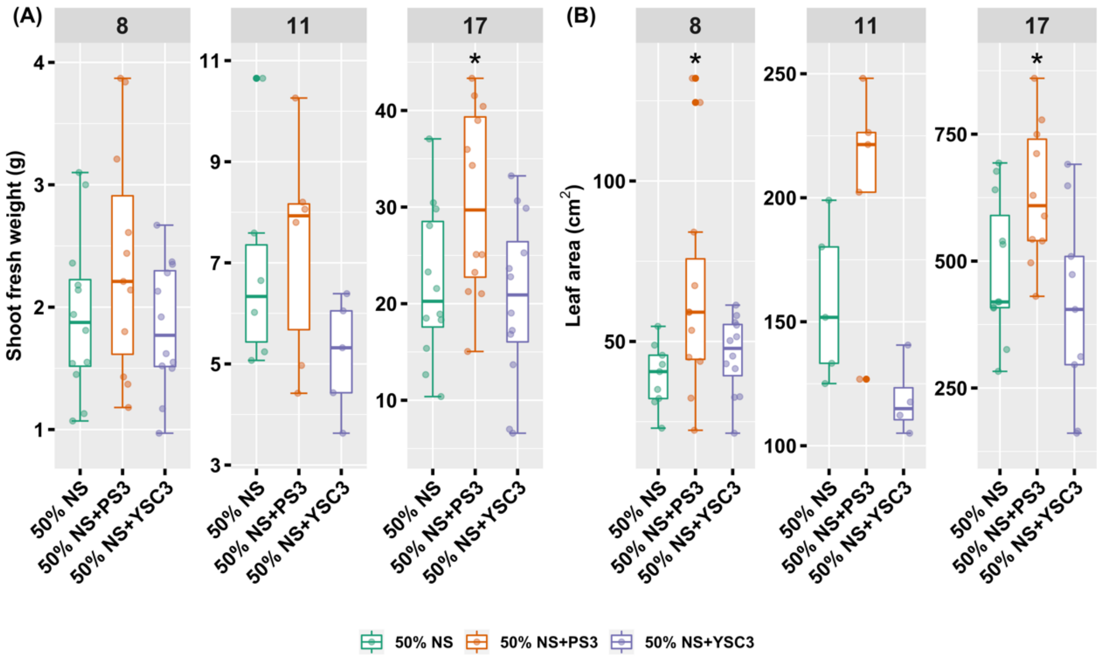

Figure S4. Beneficial effect of the PS3 strain on plant growth (third bioassay). The fresh weights of shoots **(A)** and total leaf area **(B)** were measured at 8, 11 and 17 days after treatment. Box plots show the median (horizontal bar within box) and the whiskers extended to the first and third quantiles with individual sample points. Shoot fresh weight: n=5-12; leaf area: n= 4-12. Per timepoint, the asterisk indicates the significant differences between mock- and bacteria-treated plants according to Student's t test as in '*': $P \leq 0.05$.

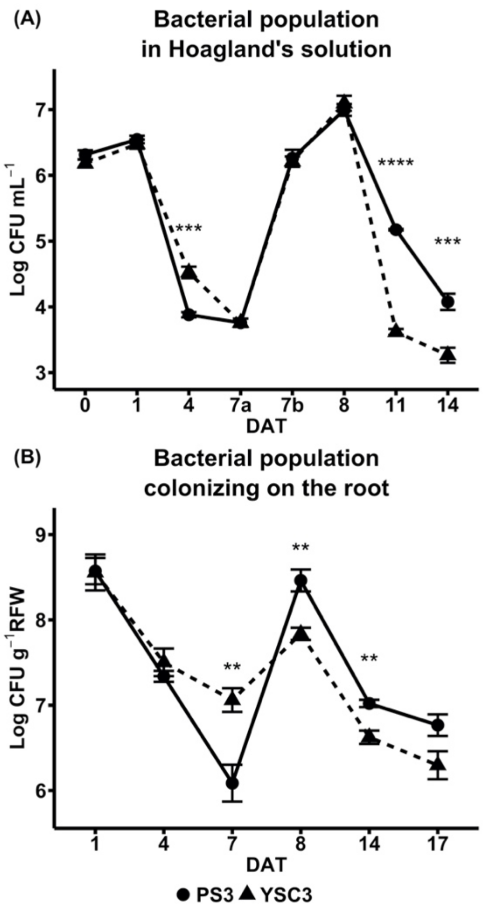

Figure S5. Bacterial population dynamics in the nutrient solution and on the root during inoculation of *R. palustris* strains in the hydroponic system. (A) Bacterial population in Hoagland's solution. Data from time point 0 represent the initial bacterial number after inoculation; time points 7a and 7b represent the bacterial population before and after the second inoculation on the 7th day after the first treatment. **(B)** The bacterial population colonizing the root during vegetation. RFW: root fresh weight. The data represent the means ± standard errors (SEs); the asterisk indicates the significant differences between PS3- and YSC3-treated plants per timepoint (Student's t test) as in '**': $P \leq 0.01$; '***': $P \leq 0.001$; '****': $P \leq 0.0001$.

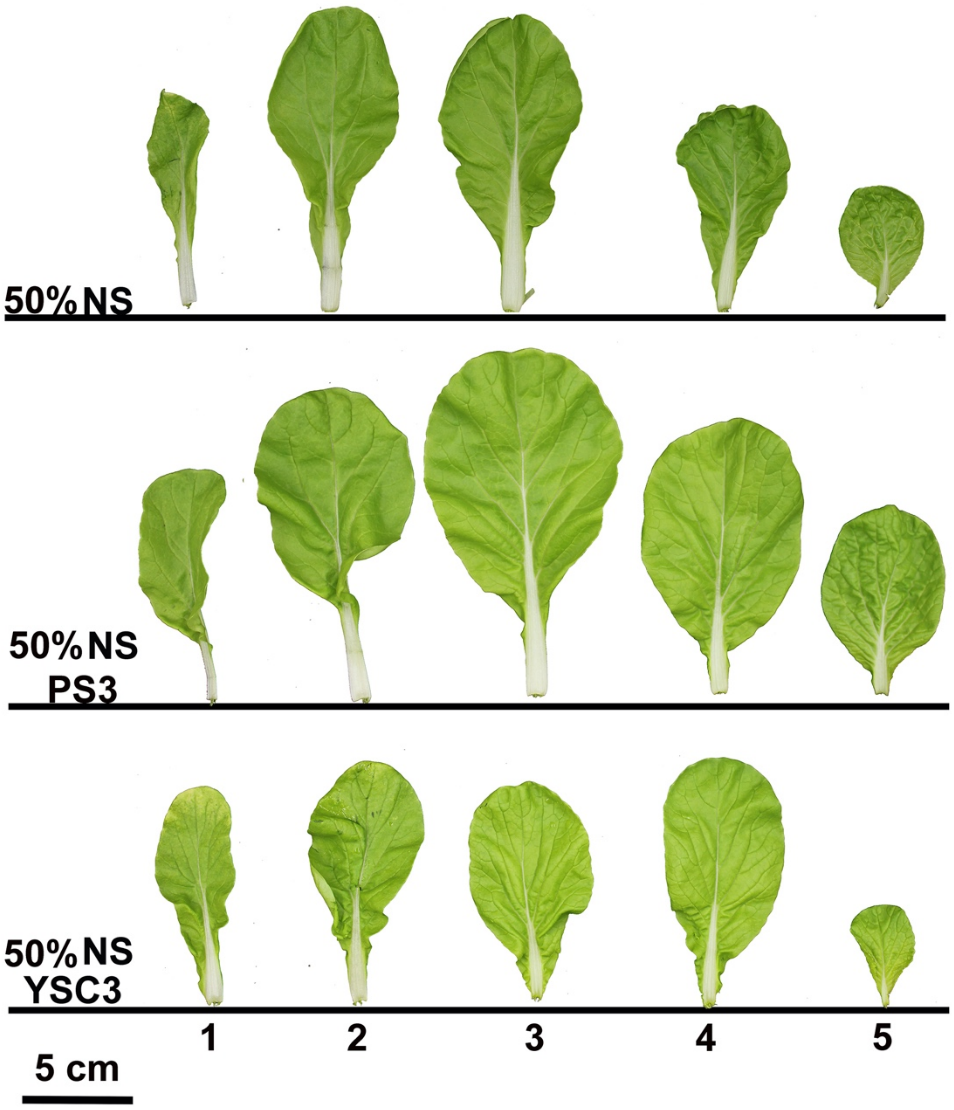

Figure S6. Changes in leaf shape in response to *R. palustris* inoculation. Plants were harvested at 15 DAT. Treatments are indicated on the left, and black scale bar at the bottom corresponds to 5 cm. The numbers at the bottom represent the positions of leaves.

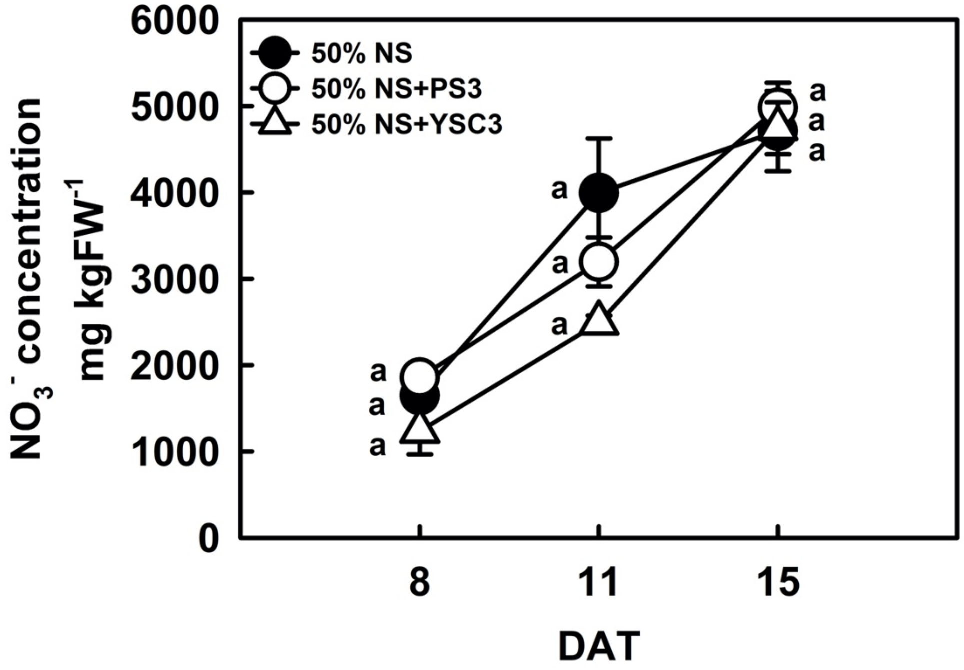

Figure S7. Changes in nitrate level in leaves in response to PS3 and YSC3 inoculation during leaf development (8, 11, 15 day after inoculation). Data are mean of four biological replicates. Error bars represent SE. Per timepoint, different letters indicate significantly differences between treatments (one-way ANOA followed by Tukey's test; $P \leq 0.05$)

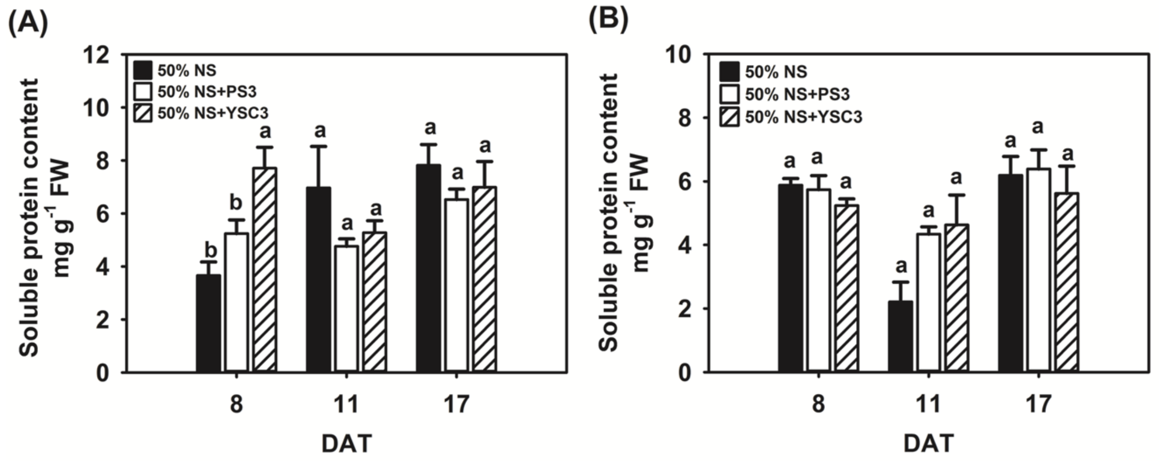

Figure S8. Soluble protein content in the leaves after inoculation with PS3 and YSC3. The soluble protein content was measured at 8, 11 and 17 DAT to estimate the nitrate reductase activity **(A)** and glutamine synthetase activity **(B)**. Bars represent means ± SEs. Per timepoint, different letters indicate significant differences between treatments (one-way ANOA followed by Tukey's test; $P \leq 0.05$; n=4)

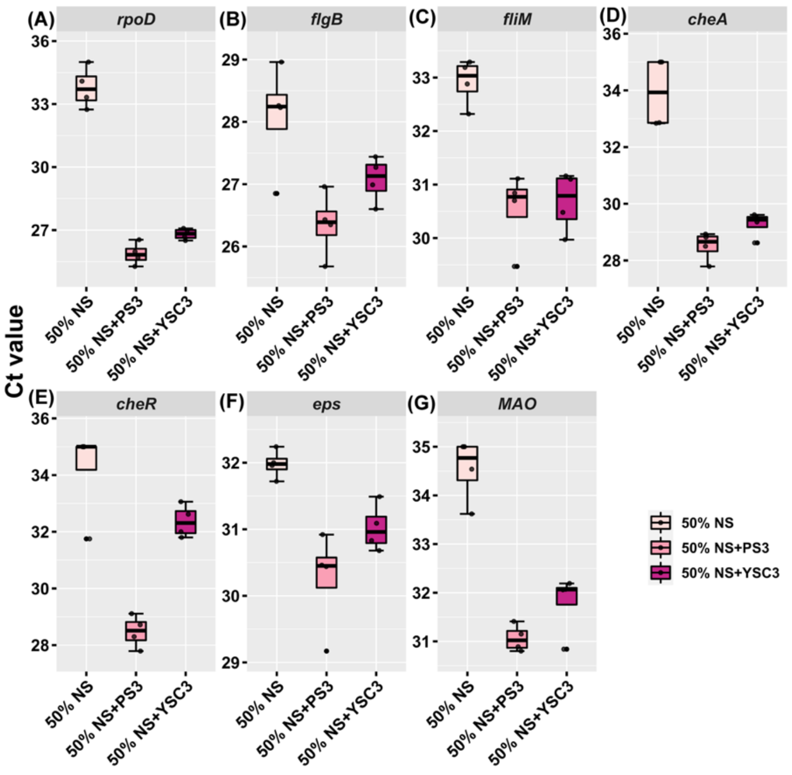

Figure S9. The threshold cycle (Ct) values of selected gene primers in the *in vivo* transcriptional profile analysis. Root-colonized bacteria were collected at 24 hours after the second inoculation (8 DAT) from non-inoculated treatment (50% NS), PS3-inoculated treatment (50% NS+PS3) and YSC3-inoculated treatment (50% NS+YSC3) plants. Ct values of **(A)** *rpoD,* **(B)** *flgB*, **(C)** *fliM*, **(D)** *cheA*, **(E)** *cheR*, **(F)** *eps* and **(G)** *MAO* gene primer sets for each treatment were determined by quantitative RT-PCR. Box plots show the median (horizontal bar within box) and the whiskers extended to the first and third quantiles. Each boxplot represents four biological repeats consisting of bacteria from three plants root per biological repeat.

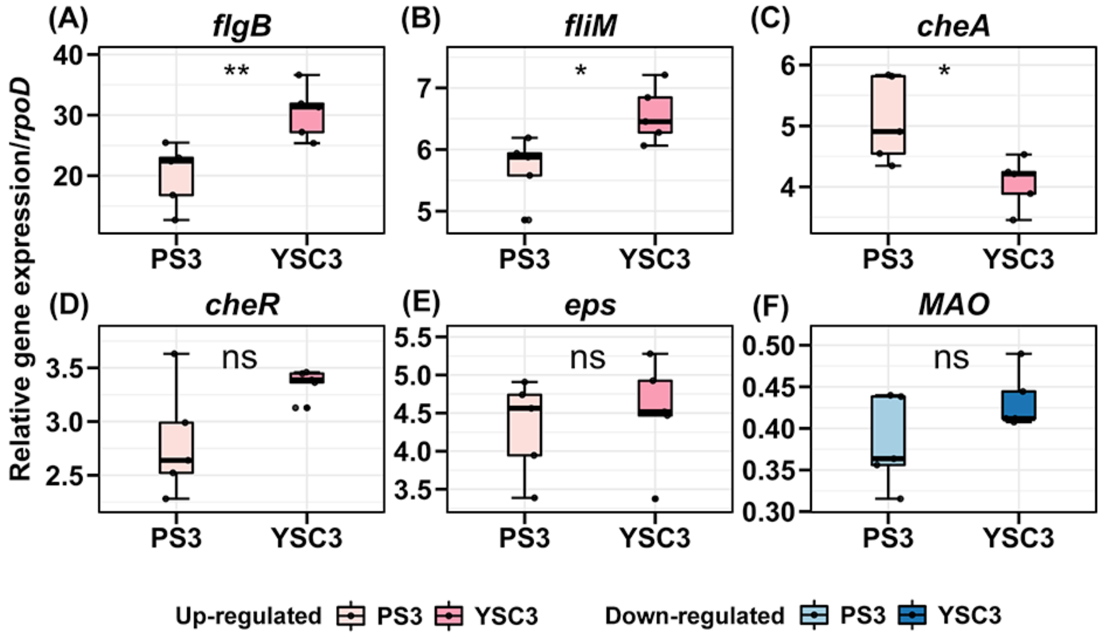

Figure S10. Gene expression patterns of *R. palustris* PS3 and YSC3 in response to half strength Hoagland's solution. PS3 and YSC3 were inoculated (10%, v/v) into 30 mL of half strength Hoagland's solution and incubated at 25 ^{o}C and 220 rpm in the dark. Bacterial cells were collected after 24 hours incubation. The expression pattern of genes implicated in **(A, B)** flagellar motility (*flagB, fliM*), **(C, D)** chemotaxis (*cheA, cheR*), **(E)** biofilm formation (*eps*) and **(F)** IAA biosynthesis (*MAO*) were determined by quantitative RT-PCR. Gene expression was normalized to that of reference gene *rpoD*. Box plots show the median (horizontal bar within box) and the whiskers extend to the first and third quantiles. Each boxplot represents five biological repeats. The asterisks indicate the significant differences between PS3- and YSC3 strains according to Student's t test, as in 'ns': $P > 0.05$; '*': $P \leq 0.05$; '**': $P \leq 0.01$.
